# A Competition of Critics in Human Decision-Making

**DOI:** 10.5334/cpsy.64

**Published:** 2021-08-12

**Authors:** Enkhzaya Enkhtaivan, Joel Nishimura, Cheng Ly, Amy L. Cochran

**Affiliations:** 1 Department of Mathematics, University of Wisconsin, Madison, WI, US; 2 School of Mathematical and Natural Sciences, Arizona State University, Glendale, AZ, US; 3 Department of Statistical Sciences and Operations Research, Virginia Commonwealth University, Richmond, VA, US; 4 Department of Population Health Sciences, University of Wisconsin, Madison, WI, US

**Keywords:** Decision-Making, Reward Learning, Computational Model, Serotonin, Reaction Time, Risk

## Abstract

Recent experiments and theories of human decision-making suggest positive and negative errors are processed and encoded differently by serotonin and dopamine, with serotonin possibly serving to oppose dopamine and protect against risky decisions. We introduce a temporal difference (TD) model of human decision-making to account for these features. Our model involves two critics, an optimistic learning system and a pessimistic learning system, whose predictions are integrated in time to control how potential decisions compete to be selected. Our model predicts that human decision-making can be decomposed along two dimensions: the degree to which the individual is sensitive to (1) risk and (2) uncertainty. In addition, we demonstrate that the model can learn about the mean and standard deviation of rewards, and provide information about reaction time despite not modeling these variables directly. Lastly, we simulate a recent experiment to show how updates of the two learning systems could relate to dopamine and serotonin transients, thereby providing a mathematical formalism to serotonin’s hypothesized role as an opponent to dopamine. This new model should be useful for future experiments on human decision-making.

## Introduction

Temporal difference (TD) learning has enjoyed tremendous support as a conceptual framework for understanding how people make decisions and what might be computed in the brain. TD learning is also supported by studies suggesting that prediction errors derived from a TD model are encoded in dopamine transients (Cohen, Haesler, Vong, Lowell, & Uchida, 2012; Montague, Dayan, & Sejnowski, 1996; Pan, Schmidt, Wickens, & Hyland, 2005; Schultz, Apicella, & Ljungberg, 1993; Schultz, Dayan, & Montague, 1997; Zaghloul et al., 2009). Recent theories and experiments, however, suggest that TD models can oversimplify human decision-making in meaningful ways (Dabney et al., 2020; Daw, Kakade, & Dayan, 2002; Kishida et al., 2016; Moran et al., 2018). In particular, models that are sensitive to risk or track multiple errors are better able to predict what decisions a person selects (Cazé & van der Meer, 2013; Chambon et al., 2020; d’Acremont, Lu, Li, Van der Linden, & Bechara, 2009; Gershman, Monfils, Norman, & Niv, 2017; Hauser, Iannaccone, Walitza, Brandeis, & Brem, 2015; Jepma, Schaaf, Visser, & Huizenga, 2020; Lefebvre, Lebreton, Meyniel, Bourgeois-Gironde, & Palminteri, 2017; Li, Schiller, Schoenbaum, Phelps, & Daw, 2011; Niv, Edlund, Dayan, & O’Doherty, 2012; Preuschoff, Quartz, & Bossaerts, 2008; Redish, Jensen, Johnson, & Kurth-Nelson, 2007; Ross, Lenow, Kilts, & Cisler, 2018; Yu & Dayan, 2005), yet the brain structures involved are not completely known. Similarly, a single-neurotransmitter based circuit, where positive concentrations match prediction-error, would struggle to encode large negative updates (Niv et al., 2012). Indeed, recent evidence suggests that serotonin may play a complementary role (Cools, Nakamura, & Daw, 2011; d’Acremont et al., 2009; Daw et al., 2002; J. Deakin, 1983; J. W. Deakin & Graeff, 1991; Montague, Kishida, Moran, & Lohrenz, 2016; Moran et al., 2018; Preuschoff et al., 2008; Rogers, 2011), though this hypothesis is still being debated. Our goal was to develop and analyze a simple computational model that resolves and unites these observations. Our proposed model involves dual critics, composed of an optimistic dopamine-like TD learner and a pessimistic serotonin-like TD learner, who compete in time to determine decisions.

TD learning was designed to utilize simple mathematical updates to produce a system that learns how to make decisions (Sutton & Barto, 2018). Such models decompose decision-making into two processes: a learning process, which updates how one values a decision, and a decision process, which selects decisions according to how they are valued. These models, including the model of Rescorla and Wagner (Rescorla & Wagner, 1972), can learn about reward expectations through updates that are linear in a single prediction error, but are not sensitive to risk or track multidimensional errors.

One reason to expect risk-sensitivity is there is asymmetry in how negative versus positive errors are updated. Dopamine transients, for example, have been found to respond more greatly to positive prediction errors than negative prediction errors (Bayer & Glimcher, 2005). From a biological perspective, this is not surprising. Dopamine neurons have low baseline activity, which imposes a physical limit on how much their firing rates can decrease because firing rates are non-negative (Niv, Duff, & Dayan, 2005). This limit suggests that dopamine neuron firing rates could not be decreased to encode negative prediction errors to the same degree as they can be increased to encode positive prediction errors. If this is true, then the outsized influence of positive prediction errors would inflate the valuation of decisions — colloquially referred to as “wearing rose-colored glasses.”

Computational models capture risk-sensitivity by weighing positive prediction errors differently than negative prediction errors, usually accomplished with separate learning rates for positive and negative prediction errors. These models are referred to as *risk-sensitive*, because they result in decision-making that is sensitive to large gains (i.e. *risk-seeking*) or large losses (i.e, *risk-averse*). Taken to an extreme, risk-seeking involves pursuing best possible outcomes, whereas risk-aversion involves avoiding worse possible outcomes (Mihatsch & Neuneier, 2002). For comparison, traditional TD learning is considered *risk-neutral* because it focuses on maximizing average (long-term discounted) rewards, so that all rewards, regardless of size, are weighted equally. Risk-sensitive models are frequently found to fit data better than risk-neutral models (Chambon et al., 2020; Hauser et al., 2015; Lefebvre et al., 2017; Niv et al., 2012; Ross et al., 2018). Importantly, differences in risk-sensitivity, substantiated by a risk-sensitive learning model, is thought to underlie certain differences between individuals with and without psychiatric disorders (Korn, Sharot, Walter, Heekeren, & Dolan, 2014; Rouhani & Niv, 2019).

The multidimensional aspect of TD-based human decision-making is supported by recent studies. Although there is no consensus about serotonin’s role in decision-making, one theory is that serotonin also encodes prediction errors but acts as an opponent to dopamine (Daw et al., 2002; Moran et al., 2018). In Moran et al, for example, serotonin transients were found to respond to prediction errors in an opposite direction of dopamine transients (Moran et al., 2018). Their results were consistent with the hypothesis that serotonin protects against losses during decision-making (Moran et al., 2018) or more broadly, plays a role in avoidance behavior (Dayan & Huys, 2008, 2009; J. Deakin, 1983). Furthermore, a recent study even suggests dopamine is capable of capturing a distribution of prediction errors, the computational benefit of which is that the reward distribution can be learned rather than just its average and variance (Dabney et al., 2020). Other conceptual frameworks suggest individuals keep track of multiple prediction errors as a way to capture the standard deviation of rewards in addition to expected rewards (Gershman et al., 2017; Jepma et al., 2020; Li et al., 2011; Redish et al., 2007; Yu & Dayan, 2005).

In this paper, we introduce and analyze a new model of human decision-making, which we call the Competing-Critics model, which uses asymmetrical and multidimensional prediction errors. Based on a TD learning framework, the model decomposes decision-making into learning and decision processes. The learning process involves two competing critics, one optimistic and another pessimistic. The decision process integrates predictions from each system in time as decisions compete for selection. In what follows, we explore through simulation whether our model can capture ranges of risk-sensitive behavior from risk-averse to risk-seeking and can reflect reward mean and variance. Further, we use this model to make predictions about reaction times and about uncertainty-sensitivity in terms of the degree to which the standard deviation of rewards influences a person’s consideration of multiple decisions. Lastly, we show how prediction errors in the Competing-Critics model might relate to dopamine and serotonin transients in the experiments of Kishida *et al* (Kishida et al., 2016) and Moran *et al* (Moran et al., 2018). Considering the simplicity of this model and its ability to synthesize several theories and experimental findings, this model should be useful as a framework for future human decision-making experiments, with potential to provide both predictive power and mechanistic insight.

## Modeling

**Figure 1:**
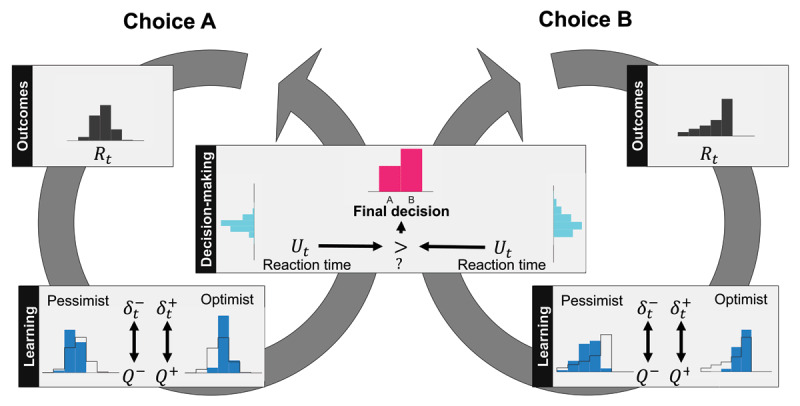
High-level view of proposed model in an example with two choices. For each choice, the distribution of rewards *R_t_* (gray histograms) is learned by competing critics through the updates *δ_t_^+^* and *δ_t_^–^*. One system is optimistic, upweighting large rewards, and another is pessimistic, downweighting large rewards (blue histograms). As a result, each choice is associated with multiple values *Q*^–^ and *Q*^+^. To determine which choice is selected, a random variable *U_t_* is drawn for each choice uniformly from (*Q*^–^,*Q*^+^) (teal histograms). The largest *U_t_* determines which choice is selected and when the decision is made.

We introduce a model of human decision-making that relies on two competing learning systems. ***Figure 1*** provides a high-level view of the proposed model in a simple example in which an individual makes decisions between two choices. Here the individual learns to value their decision by weighing prior outcomes observed upon selecting each choice, denoted by *R_t_*, in two different systems. The first learning system weighs better outcomes more heavily than worse outcomes, which effectively leads to a more optimistic valuation of outcomes, denoted by *Q^+^*. The second learning system does the opposite: weighs worse outcomes more heavily than better outcomes, leading to a more pessimistic valuation of outcomes, denoted by *Q^–^*. We remark that both values, *Q^+^* and *Q^–^*, are assumed to be updated according to prediction errors *δ_t_^+^* and *δ_t_^–^*, following common risk-sensitive temporal difference (TD) learning frameworks described below.

An individual who relied solely on the first learning system to make decisions would be considered *risk-seeking* due to the outsized influence of better outcomes. Similarly, an individual who relied solely on the second system to guide decisions would be considered *risk-averse* due to the outsized influence of worse outcomes. Our model, however, supposes both of these competing learning systems contribute to decision-making in the following way. For each choice, the risk-seeking learning system sends a *go* signal to the individual to signify that this choice is viable, with larger *Q^+^* values corresponding to earlier signals. Afterwards, the risk-sensitive learning system sends a *no-go* signal to the individual to signify that this choice is no longer viable, with smaller *Q^–^* associated with later signals. For simplicity, the individual is assumed to select the respective choice at any time between these two signals, provided no other choice has been selected or choice exploration has been pursued. Hence, both go and no-go signals determines how likely each choice is selected. For example, choices whose go signal is initiated after a no-go signal of another choice will never be selected except for exploration. Put differently, any choice when valued optimistically is still worse than another choice valued pessimistically will not be selected except for exploration. We now proceed to formalize this conceptual framework.

### Setting

Our model will describe psychological experiments that have the following decision-making scenario. The scenario starts at the initial state *S*_0_ on which the participant bases their action *A*_0_, which brings in a numerical reward *R*_1_. Consequently, the participant finds itself in the next state *S*_1_ and selects another action *A*_1_, which brings in a numerical reward *R*_2_ and state *S*_2_. This process then repeats until the participant makes *T* decisions, yielding a sequence of observations collected for each participant of the form:


\[
{S_0},\ {A_0},\ {R_1},\ {S_1},\ {A_1},\ {R_2},\ {S_2},\  \ldots,\ {R_{T-1}},\ {S_{T- 1}},\ {A_{T-1}},\ {R_T}.
\]

Above, observations fall into three types on a given trial *t*: the *state* that the participant visits, denoted by *S_t_*, the *action* that the participant takes when visiting state *S_t_*, denoted by *A_t_*, and the subsequent *reward, R_t+_*_1_, that a participant receives upon visiting state *S_t_* and taking action *A_t_*. For simplicity, let us assume that both the space of possible states \[
{\mathcal{S}}
\] and the space of possible actions \[
{\mathcal{A}}
\] are discrete. The space of possible rewards \[
{\mathcal{R}}
\] can be any subset of the real line ℝ. Further, assume the experiment defines subsequent rewards and states as a function of the current state and action according to a Markov transition probability


\[
p\left( {{s^{\prime}},r|s,a} \right): {\cal S} \times {\cal R} \times {\cal S} \times {\cal A} \to {\mathrm{ }}\left[ {0,1} \right].
\]

An experiment described above constitutes a (discrete-time, discrete-state) Markov Decision Process (MDP).

### Temporal difference (TD) learning

In the setting described above, human decision-making is often modeled using TD learning. One widely-known algorithm for TD learning is called Q-learning, so-named for its explicit use of a state-action value function denoted by *Q*. This algorithm supposes that the agent, i.e., the participant in a psychological experiment, tries to learn the “value” of their actions as a function of a given state in terms of future rewards. This notion gives rise to a state-action value function *Q*(*s,a*) mapping states *s* ∈ \[
{\mathcal{S}}
\] and actions *a* ∈ \[
{\mathcal{A}}
\] to a real number that reflects the value of this state-action pair. A Q-learner updates this state-action function according to their experiences:


1 \[
Q({S_t},\:{A_t}) \leftarrow Q({S_t},\:{A_t}) + \alpha \left[ {{R_{t + 1}} + \gamma \:\mathop {\max }\limits_a \:Q({S_{t + 1}},\:a) - Q({S_t},\:{A_t})} \right].
\]

Here, the learner has just taken action *A_t_* in state *S_t_*, receiving the immediate reward *R_t_*_+1_ and transitioning to a new state *S_t_*_+1_. A learning rate *α* accounts for the extent to which the new information, i.e. their reward and the new state-action value, overrides old information about their state-action value function. For instance, one can see that if *α* = 0, there is no overriding - the estimate stays the same. The discount parameter *γ* weighs the impact of future rewards. A discount parameter *γ* = 0 would mean the learner does not care about the future at all, while *γ* = 1 would mean the learner cares about the sum total of future rewards (which may even cause the algorithm to diverge).

### Risk-sensitive TD learning

A variant of the Q-learner allows a learner to be particularly sensitive to smaller, or more negative, rewards, i.e. *risky* situations. In particular, a risk-sensitive Q-learner weighs the prediction error, which is given by


\[
{\delta _t} = {R_{t + 1}} + \gamma \;\mathop {\max }\limits_a \;Q({S_{t + 1}},\;a) - Q({S_t},\;{A_t}),
\]

differently depending on whether the prediction error is positive or negative. This yields the following update:


\[
Q({S_t},\;{A_t}) \leftarrow Q({S_t},\;{A_t}) + \alpha \left[ {(1 + k){\mathrm{ }}{1_{{\delta _t} > 0}} + (1 - k){\mathrm{ }}{1_{{\delta _t} < 0}}} \right]{\delta _t}.
\]

The parameter *k* controls the degree to which the learner is risk sensitive. If *k* = 0, then the learner weighs positive and negative prediction errors equally, in which the updates are the same as before and we say the learner is *risk-neutral*. If *k* < 0, then negative prediction errors are weighed more than positive prediction errors. In this case, smaller rewards have a stronger influence relative than larger rewards on the state-action value function *Q*, resulting in a learner who is considered *risk-averse*. Similarly if *k* > 0, the reverse is true: larger rewards have a stronger influence relative to smaller rewards, and the learner is considered *risk-seeking*.

### A learning model with competing critics

With the introduction of risk-sensitive TD learning, we can consider a range of learning behaviors from risk-sensitive to risk-seeking, all modulated by parameter *k* and reflected in the state-action value function *Q*. Researchers are often focused on how pessimism or risk-sensitivity, substantiated by *k*, might vary between individuals. In our model, however, we investigate how risk-sensitivity might vary within individuals. Specifically, we consider two learning systems, one pessimistic (risk-adverse) and one optimistic (risk-seeking).

Our model captures two competing critics by keeping track of two state-action value functions, *Q^+^* and *Q^–^*, and updated each function according to:


2 \[
\begin{array}{*{20}{c}}
{{Q^ + }({S_t},\;{A_t}) \leftarrow {Q^ + }({S_t},\;{A_t}) + \alpha \left[ {(1 + {k^ + }){1_{\delta _t^ + > 0}} + (1 - {k^ + }){1_{\delta _t^ + < 0}}} \right]\delta _t^ + }\\
{{Q^ - }({S_t},\;{A_t}) \leftarrow {Q^ - }({S_t},\;{A_t}) + \alpha \left[ {(1 - {k^ - }){1_{\delta _t^ - > 0}} + (1 + {k^ - }){1_{\delta _t^ - < 0}}} \right]\delta _t^ - }
\end{array}
\]

with prediction errors given by


\[
\begin{array}{*{20}{c}}
{\delta _t^ + = {R_{t + 1}} + \gamma \;\mathop {\max }\limits_a \;{Q^ + }({S_{t + 1}},\;a) - {Q^ + }({S_t},\;{A_t})}\\
{\delta _t^ - = {R_{t + 1}} + \gamma \;\mathop {\max }\limits_a \;{Q^ - }({S_{t + 1}},\;a) - {Q^ - }({S_t},\;{A_t}).}
\end{array}
\]

For simplicity, we initialize *Q*^+^ and *Q*^–^ to zero. Parameters *k^+^,k*^–^ are assumed to lie in [0, 1]. Large *k*^+^ controls the degree to which the learner is risk-seeking and *k*^–^ controls the degree to which the learner is risk-sensitive. It is important to point out that we are also not the first to consider multiple risk-sensitive TD learning systems. This idea was recently put forth in (Dabney et al., 2020), where multiple risk-sensitive TD learning systems were thought to be encoded in multiple dopamine neurons. We are also not the first to consider dual competing systems (Collins & Frank, 2014; Daw et al., 2002; Mikhael & Bogacz, 2016; Montague et al., 2016). In the opposing actor learning model in (Collins & Frank, 2014), for example, prediction error from a single learning system controls the dynamics of G (“go”) and N (“no-go”) systems, which in *turn* are combined linearly to determine decisions. Since it may not be obvious why this model differs from our proposed model, we discuss in the Supplement how the update equations of the two models differ in important ways, resulting in significantly different behaviors and predictions. Similarly, the Supplement also explores differences between our proposed model and a SARSA version of the model as well as a risk-sensitive TD learning model.

### A decision-making model with competing critics

Now that we have a model of learning, namely *Q*^+^ and *Q*^–^, it is sensible to consider how the agents makes decisions based on what they have just learned. This means that the individual has to make the decision of choosing from the available actions, having obtained pessimistic and optimistic estimates for action-value pairs.

A naive approach is what is called the greedy method, meaning that the action with the highest value is chosen. This approach, however, does not account for actions with multiple values (e.g., optimistic and pessimistic values) nor does it allow the individual to do any exploration, during which they might discover a more optimal strategy. A way to incorporate exploration into decision-making is to act greedy 1 – *ɛ* of the time and for *ɛ* of the time, the individual explores non-greedy action with equal probabilities. This method referred to as *ɛ*-greedy and is used by our model.

To integrate multi-valued actions into a *ɛ*-greedy method, our model supposes that a random variable *U_t_*(*a*) is selected for each action *a* uniformly from the interval [*Q*^–^(*S_t_,a*), *Q*^+^(*S_t_,a*)], whenever an individual has to make a decision in state *S_t_*. Then whenever the individual acts greedily, they select the action *A_t_* that maximizes *U_t_*(*a*). These decision rules along with learning models comprise Competing-Critics model, which is summarized in ***Algorithm 1***. While we use an *ɛ*-greedy method, exploration could also be achieved by applying a soft-max function to transform *U_t_*(*a*) into a probability and select action a according to this probability.

**Algorithm 1:** Competing-Critics.


**Input:** Learning rate *α*, parameters *k*^+^*, k*^–^, discount factor *γ*, and exploration parameter *ɛ*.
Initialize *Q*^±^(*s, a*) for all (*s,a*) ∈ \[ {\mathcal{S}} \] × \[ {\mathcal{A}} \]
Initialize S
**While** not terminated **do**
Sample *U*(*a*)∼Unif [*Q*^–^(*S, a*), *Q*^+^(*S, a*)] for each action *a* in state *S*
Choose *A* using *ɛ*-greedy from the values *U*(*a*)
Take action *A*, observe *R, S*′
*% Compute prediction errors*
*δ*^±^ ← *R*+γ max*_a_ Q*^±^(*S*′*, a*)–*Q*^±^(*S, A*)
*% Update state-action value functions*
\[ {Q^ \pm }(S,\;A) \leftarrow {Q^ \pm }(S,\;A) + \alpha \left[ {(1 \pm {k^ \pm }){1_{{\delta ^ \pm } > 0}} + (1 \mp {k^ \pm }){1_{{\delta ^ \pm } < 0}}} \right]{\delta ^ \pm } \]
*% move to new state*
*S* ← *S*′
**end while**


## Simulation Experiments

We used simulation to investigate individual behavior in several experiments were they to learn and make decisions according to our decision-making model. In particular, we wanted to identify possible vulnerabilities in behavior that arise from a shift in the balance between the internal optimist and pessimist, instantiated by changes in parameters *k*^+^ and *k*^–^. For simplicity, each simulation involves 30,000 replicates, and parameters are fixed:


\[
(\alpha ,\;\varepsilon ,\;\gamma ,\;{k^ + },\;{k^ - }) = \left( {0.5,\;0.3,\;0,\;0.9,\;0.9} \right),
\]

unless otherwise specified. In the Supplement, we also explore situations when parameters are randomly sampled to determine the degree to which any of our conclusions are sensitive to parameter choice. Further, a detailed description of the simulations can be found in the Supplement and at: *https://github.com/eza0107/Opposite-Systems-for-Decision-Making*.

### Learning the shape of rewards

Let us first focus on learning behavior by considering the simple case of trivial state and action spaces: \[
{\mathcal{S}}
\] = {1} and action \[
{\mathcal{A}}
\] = {1}. In this case, learning in the Competing-Critics model is determined completely by the distribution of rewards *R_t_*. We considered what an individual would learn given four different Pearson distributions of *R_t_*, with varying mean *μ*, standard deviation *σ*, and skew, while kurtosis was fixed at 2.5. For reference, we also consider the classic *Q* described at Eq. (1).

**Figure 2:**
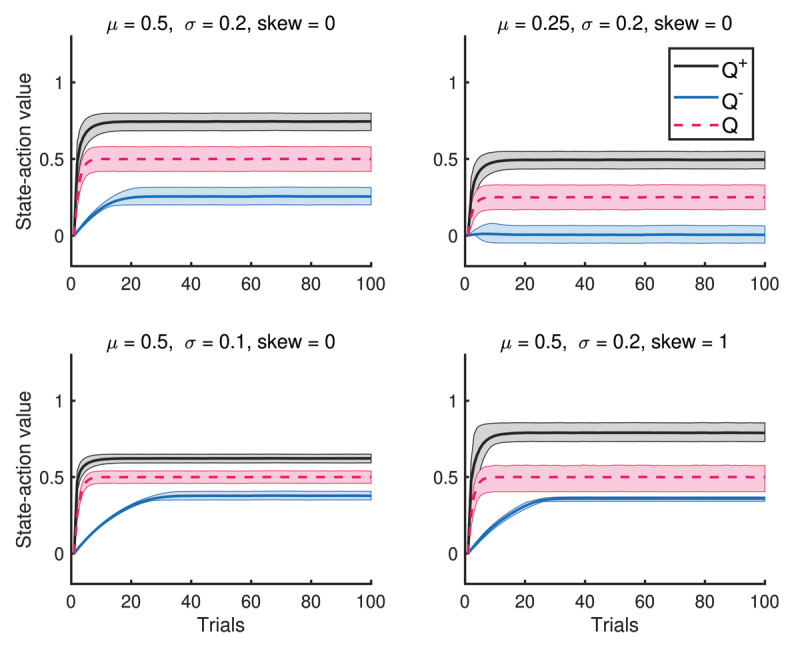
Comparison of mean and interquartile range of state-action value functions over 30,000 simulations. The state-action values *Q*^+^ and *Q*^–^ reflect changes in the mean *μ*, standard deviation *σ*, and skew of the reward distribution. Notably, asymptotes of these values shift by 0.25 when *μ* decreases by 0.25, and their gap decreases by 1/2 when *σ* decreases by a factor of 1/2.

***Figure 2*** illustrates what an individual with balanced parameters, (*k*^+^, *k*^–^) = (0.9, 0.9), learns over 100 trials. For comparison, we also simulated a traditional, risk-neutral *Q* learning model by setting *k*^+^ *= k*^–^ = 0. Solid dark lines denote state-action value function averaged over simulations and shaded regions represent associated interquartile ranges (IQRs) for each function. One can immediately notice several things. By design, the optimistic value function *Q*^+^ is on average larger than the neutral value function *Q*, which is larger than the average pessimistic value function *Q*^–^. In addition, the distribution of each value function appears to converge and can capture shifts in mean rewards μ and scaling of the standard deviation *σ*. Specifically, the long-term relationship between *Q*^+^*,Q*^–^ and *Q* is preserved when *μ* is shifted from 0.5 to 0.25, whereby all value functions shift down by about 0.25. Further, the gap between *Q*^+^ and *Q*^–^ is halved when *σ* is halved from 0.2 to 0.1; each IQR is also halved. Meanwhile, *Q*^+^ and *Q*^–^ are roughly symmetric around the *Q* when the reward distribution is symmetric (i.e. zero skew), so that the average of *Q*^+^ and *Q*^–^ is approximately *Q*. However, moving skew from 0 to 1 is reflected in both the gap between *Q*^+^ and *Q*, which lengthens, and the gap between *Q*^–^ and *Q*, which shortens.

**Figure 3:**
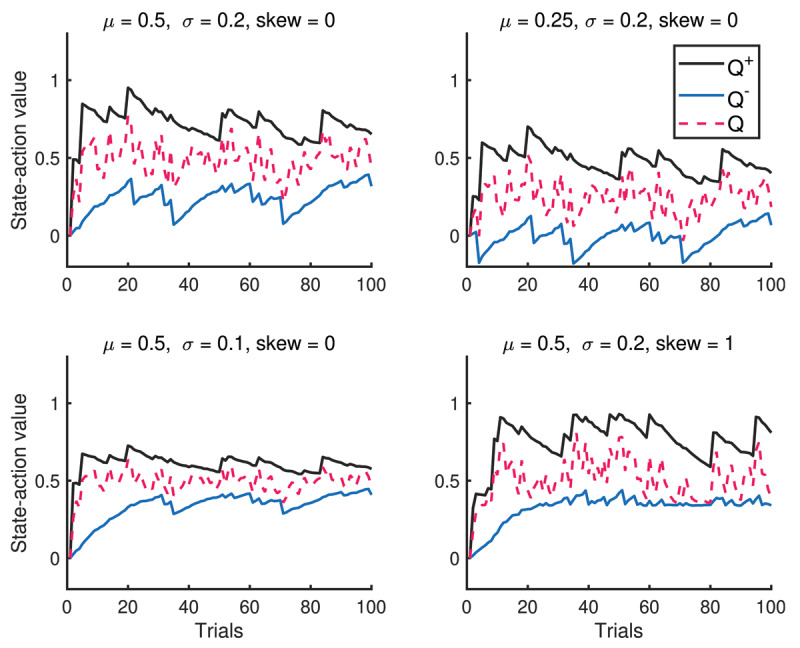
A single simulation run of state-action value functions *Q*^±^ and *Q*. The state-action values preserve the ordering *Q*^–^< *Q*< *Q*^+^ through the entire run.

Remarkably, the relationship *Q*^+^ > *Q* > *Q*^–^ is also present within a single simulation run (***Figure 3***). Intuitively, this makes sense because they capture the behaviors of risk-seeking, risk-neutral and risk-sensitive agents, respectively and it turns out that this ordering can be preserved provided k^±^ are neither too small or large. See Supplement for the proof of this result. Furthermore, the last subplot also illustrates that introducing a positive skew to the reward distribution *R_t_*, also causes the distribution of *Q*^±^ and *Q* to also have positive skew.

**Figure 4:**
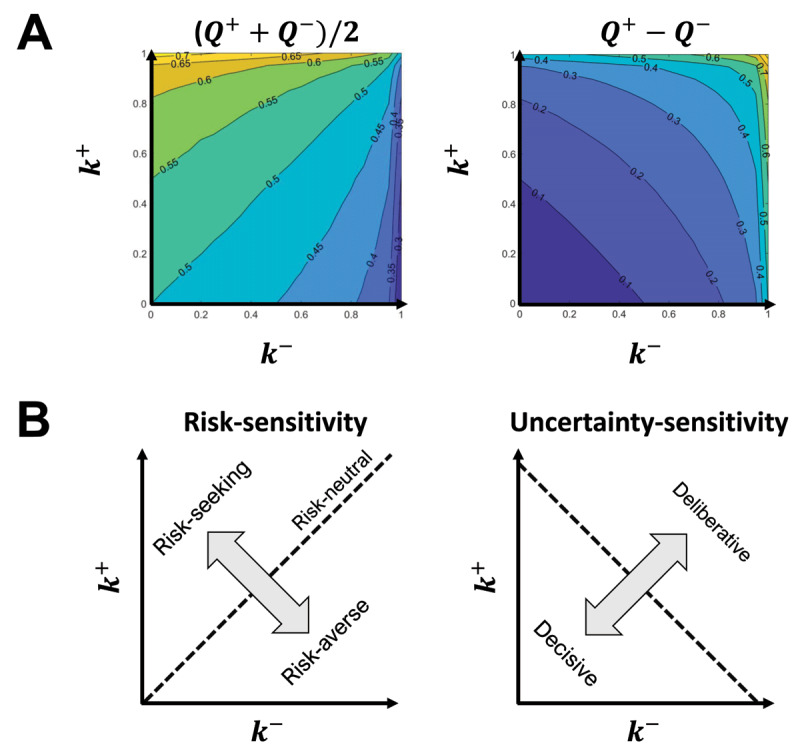
Impact of parameters *k*^+^ and *k*^–^ on **A)** the midpoint and gap between *Q*^+^ and *Q*^–^ averaged over 30,000 simulations, and **B)** how an individual makes decisions. In particular, the model decomposes decision-making behavior along two axes, a risk-sensitivity and an uncertainty-sensitivity, which are rotated 45° degrees from the *k*^±^ axes. In the simulation, *μ* = 0.5, *σ* = 0.2, and skew = 0.

Value functions *Q*^+^ and *Q*^–^ are not only modulated with the reward distribution, but also parameters *k*^±^. Increasing *k*^+^ moves *Q*^+^ in a positive direction away from the risk-neutral value function *Q*, whereas increasing *k*^–^ moves *Q*^–^ in a negative direction away from the risk-neutral value function *Q*. With *k*^+^ pulling *Q*^+^ in one direction and *k*^–^ pulling *Q*^–^ in the opposite direction, the midpoint of *Q*^+^ and *Q*^–^ is largely influenced by the gap in *k*^–^ and *k*^+^ (***Figure 4A***). Meanwhile, the gap between *Q*^+^ and *Q*^–^ is largely influenced by the midpoint of *k*^+^ and *k*^–^.

**Figure 5:**
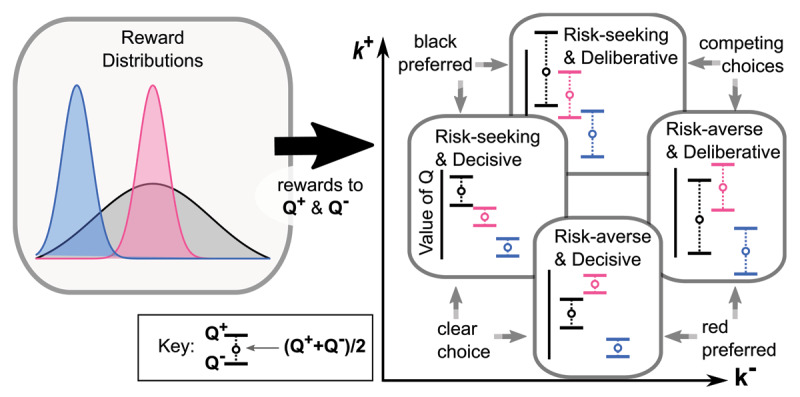
Four different decision makers with different *k*^+^ and *k*^–^ parameter values interpret the same reward distributions differently. Parameter values associated with risk-seeking are more likely to prefer the rewards drawn from the black distribution, while risk-averse parameter values prefer the red distribution. Meanwhile, deliberative parameter values are more likely to explore the two best competing choices, as those choices have overlap between their *Q* intervals, while decisive parameter values pick only their preferred distribution. Note that none of the four learners would select the blue distribution.

Thus, while *k*^+^ and *k*^–^ are the two natural parameters of the learning process, the difference in how agents make choices is well described by a 45° rotation of these coordinates, yielding axes *s_r_* = *k*^+^ – *k*^–^ and *s_u_* = *k*^+^ + *k*^–^. As visualized in ***Figure 4B***, we refer to the *s_r_* and *s_u_* axes as the risk-sensitivity and uncertainty-sensitivity axes, respectively. These two axes provide orthogonal ways of interpreting and comparing different reward distributions, as in ***Figure 5***. Namely, risk-sensitivity, which can vary from risk-averse to risk-seeking, captures a learner’s bias either against losses or towards gains, and is instantiated in our model as the difference between \[
{\textstyle{1 \over 2}}({Q^ + } + {Q^ - })
\] and the expected reward. In contrast, uncertainty-sensitivity, which can vary from decisive to deliberative, captures a learner’s consideration of actions with large standard deviations in rewards. In our model, this uncertainty-sensitivity is instantiated as the size of the interval between *Q*^–^ and *Q*^+^, wherein the larger that interval, the more likely two actions with similar values of \[
{\textstyle{1 \over 2}}({Q^ + } + {Q^ - })
\]2 are to be seen as competing, viable choices.

While increasing uncertainty-sensitivity can increase the variety of actions that a learner makes, it is distinct from the standard use of an exploration parameter ∈. An exploration parameter ∈ forces the exploration of all possible actions, and is included to ensure that no action is left unexplored. By contrast, uncertainty-sensitivity is a preference axis, and it only encourages the exploration of competitive actions whose intervals overlap with the action with the largest value \[
{\textstyle{1 \over 2}}({Q^ + } + {Q^ - })
\] . The preference aspect of uncertainty-sensitivity is especially clear in cases where many actions with high variance rewards are considered against a single reliable action with a fixed outcome (no variance) and a slightly higher expected reward. In such a setting, a deliberative learner may often pick the high variance actions even though they could correctly report that the fixed outcome had a better expected outcome (by contrast, a risk-seeking learner would report the high variance actions as having better outcomes). Indeed, while both risk-sensitivity and uncertainty-sensitivity can describe why a learner might prefer a high variance reward to a fixed reward with slightly higher expected return, both are required to explain why some learners might exclusively choose the high variance action while some others sample both the high variance action and the fixed outcome. Similarly, the difference between uncertainty and risk-sensitivity can affect the choices when a fixed outcome would preferred, as also illustrated in ***Figure 5***.

In summary, parameters *k*^±^ can capture a range of behavior from being too risky to not risky enough and from too decisive to too deliberative. We demonstrate these decision-making behaviors in the next two examples.

### Capturing a penchant for gambling

To demonstrate how parameters *k*^±^ drive decision-making behavior in our model, let us consider the Iowa Gambling Task (IGT), which asks participants to repeatedly chose between four decks labeled A to D. After each choice, they gain and/or lose monetary rewards. Hundreds of studies have used the IGT to evaluate human decision-making (Chiu, Huang, Duann, & Lin, 2018). Initial findings found healthy controls would learn to select “good” decks (Decks C and D), so-called because, on average, they yielded a net gain (Bechara, Damasio, Damasio, & Anderson, 1994). By contrast, individuals with a damaged prefrontal cortex would continue to select “bad” decks (Decks A and B) despite yielding net losses on average. Selecting bad decks was put forth as a marker of impaired decision-making, or more specifically, an insensitivity to future consequences. This interpretation, however, presumes that the participant’s objective is indeed to make decisions that maximize expected rewards as opposed to making decisions that seeks large gains or avoids large losses. Risk-seeking behavior (i.e. a penchant for gambling), in particular, may encourage individuals to pursue bad decks, since they yield the largest one-time gains.

The IGT can be placed with our MDP framework with *A_t_* ∈ {*A,B,C,D*} capturing the selected desks, *S_t_* ∈ {1} capturing a trivial case with only one state, and *R_t_* capturing the summed gain and loss per trial. In particular, we will simulate *R_t_* as independent draws from a distribution that depends on the selected deck and matches characteristics described in the Supplement. For example, *R_t_* is drawn uniformly from {$50, $0} when Deck C is selected.

**Figure 6:**
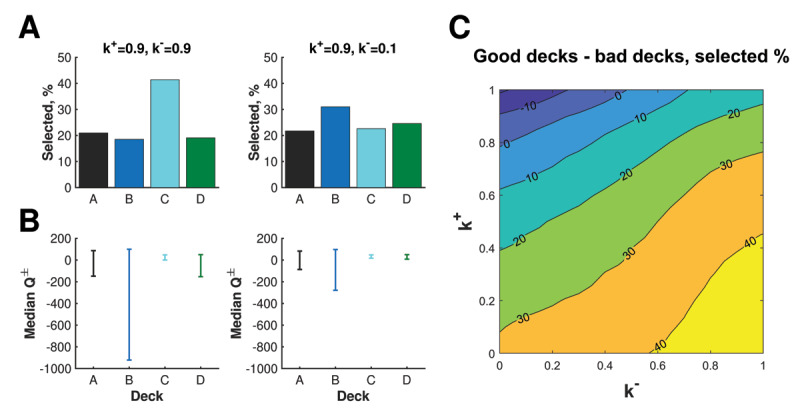
Risk-sensitivity of the Competing-Critics model during the Iowa Gambling task aggregated over 100 trials and 30,000 simulations. **A)** The “risky” Deck B becomes the most popular choice rather than Deck C, when parameter *k*^–^ is decreased from 0.9 to 0.1. **B)** Deck selection is determined by the highest value of a random variable drawn uniformly from the interval *Q*^+^ to *Q*^–^. Here, the interval from median *Q*^+^ to median *Q*^–^ is plotted to help illustrate which decks are viable options Deck B becomes more favorable because of a dramatic increase to the pessimistic value function *Q*^–^. **C)** Bad decks A and B are chosen at higher rates moving along the risk-sensitivity axis (i.e. the *k*^+^ = 1–*k*^–^ line).

To that point, balanced (*k*^+^*,k*^–^) = (0.9,0.9) parameters, reflecting risk-neutral behavior, results in a preference for Deck C, i.e. one of the good decks that leads to average net gains (Fig 6A). By contrast, imbalanced (*k*^+^*,k*^–^) = (0.9,0.1) parameters, reflecting risk-seeking behavior, results in a preference for Deck B, i.e. one of the bad decks that leads to average net losses. In each case, pessimistic state-action values *Q*^–^ are larger for good decks (C and D), correctly signifying that these decks are the more risk-averse choices (***Figure 6B***). Meanwhile, optimistic state-action values *Q*^+^ are larger for bad decks (A and B), correctly signifying that these decks are the more risk-seeking choices. Imbalanced *k*^±^ parameters, however, dramatically underplays the risk of Deck B compared to balanced risk-sensitive parameters. Consequently, the chance of large gains encoded in *Q*^+^ is suitably enticing to encourage a Deck B preference. That is, Deck B preference, which is actually a well-known phenomenon of healthy participants (Chiu et al., 2018), can be interpreted as a penchant for gambling rather than an insensitivity to future consequences.

As was done in Steingroever, Wetzels, and Wagenmakers (2013), we can also partition the parameter space {(*k*^+^, *k*^–^)| 0 ≤ *k*^+^, *k*^–^ ≤ 1} by preference for good and bad decks (Fig 6C). This figure tells us that in the “blue” region of the parameter space, bad decks A, B are selected at greater frequency than good decks C, D. In the context of risk-seeking vs risk-averse terminology, our choice of *k*^+^ >> *k*^–^ means that our learner, despite the fact that *B* incurs incomparably large loss, keeps sticking to it because *Q*^+^ is driving the choice. In another words, our agent is unable to learn the good decks in the IGT, thus mimicking the behaviors of the participants with prefrontal cortex damage as demonstrated in Lin, Chiu, Lee, and Hsieh (2007).

### The ambiguity of deliberation

One of the main conceptual insights of having two orthogonal axes of risk and uncertainty-sensitivities is that it can describe a greater variation in the types of decisions that people might make (or prefer to make) and thus allows for alternate interpretations of some experiments. To illustrate this, consider the 2-stage Markov task (Daw, Gershman, Seymour, Dayan, & Dolan, 2011), in which a participant repeatedly selects images over two stages and where the experiment was explicitly designed in order to probe the difference between model-free and model-based learning.

In the 2-stage Markov task, participants are presented one of three pairs of images at a given stage. At the first stage, all participants are shown the first pair of images and have the option to choose either the left or right image. After choosing an image, participants are shown the second or third pair of images, with the pair selected randomly according to probabilities that depends on their first stage selection. Participants then select an image on the second stage and receive monetary rewards. This task is used in experiments to determine the degree to which individuals are learning about the common (*p* = 0.7) versus rare (*p* = 0.3) transition associated with each action in stage 1. To mark this type of learning, the authors point to the probability of staying on the same first-stage decision (i.e. repeating the same first stage decision on a subsequent trial) depending on the type of transition (common vs. rare) and whether or not the person was rewarded on the second stage. In particular, the authors predicted that stay percentages of a model-free learner would differ only based on reward, while a model-based learner’s stay percentage would differ only based on whether the first transition was common or rare. In fact, the data showed that participants’ stay percentage varied by both reward and the transition type. Since neither model predicted this reward-transition interaction, the authors stated that both model-free and model-based learning are occurring.

By contrast, we believe that the observed difference in stay percentages can be well captured by our model, and that the relevant difference between the common and rare stay percentage may be capturing uncertainty-sensitivity. We model the two-stage Markov task as follows. The two-stage Markov task has actions *A_t_* ∈ {left, right} representing selected images, states *S_t_* ∈ {1,2,3} capturing presented image pairs, and rewards *R_t_* capturing rewards after image selection with rewards after the first stage set to zero. Here t counts the total number of actions. That is, *t* = 0 corresponds to the first time that a participant takes an action in the first stage, and *t* = 1 corresponds to the first time that a participant takes an action in the second stage. For our model-free model to capture reward-transition interactions, we do not distinguish between first and second stage decisions, using the same model update regardless of the decision stage. This approach effectively treats the switch from second to first stage as a state transition. To allow information to pass from between stages, we use a discount factor *γ* of 0.9. By contrast, the model-free model in Daw et al. (2011) uses different updates for first and second stage decisions and does not treat the switch from second to first stage as a state transition. Rather, they view the second stage decision as a state transition to a dummy terminal state, and subsequently rely on an eligibility trace to pass information from second to first stage decisions.

**Figure 7:**
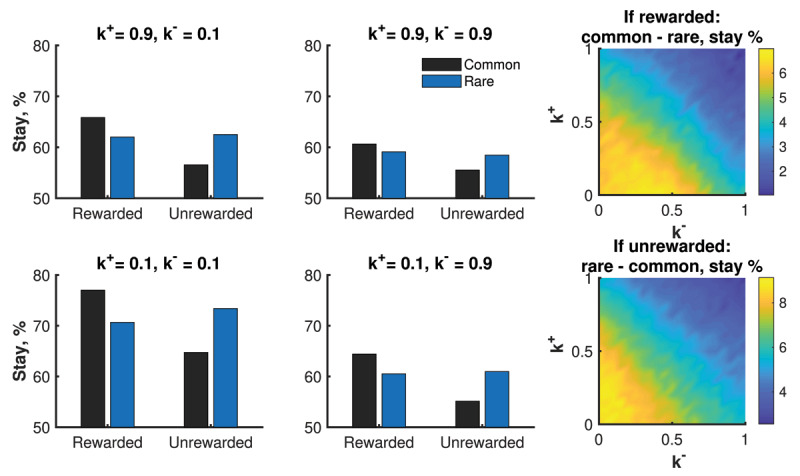
Stay probabilities after a first stage choice over a horizon of 80 decisions (40 first-stage decisions) and 30,000 simulations. The gap between stay probabilities for common vs. rarer transitions increases along the uncertainty-sensitivity axis (i.e. *k*^+^ = *k*^–^ axis) as the learner increases their deliberation about multiple choices.

The bar graphs in ***Figure 7*** represents the probability in our model of competing critics of sticking to the current choice categorized by whether it resulted in reward or not and whether the transition was common or rare. The plots on the right side of the figure tells us the difference between the probabilities of staying when the transition was common or rare, given rewarded or unrewarded.

As displayed in ***Figure 7***, the characteristic pattern observed in (Daw et al., 2011), where stay percentage depends on both rewarded/unrewarded and the common/rare transition is present with the same trends. Moreover, the degree to which there is a common/rare difference is determined by the parameters along the uncertainty-sensitivity axis (*s_u_* = *k*^–^ + *k*^+^). Namely, when *s_u_* is large, *k*^–^ = *k*^+^ = 0.9, then the model stay percentage is only slightly affected by the reward and the transition, reflecting a more deliberative sampling of actions resulting in less immediate correlations between actions in one trial and subsequent actions in the next. On the other hand, when *s_u_* is small, *k*^–^ = *k*^+^ = 0.1, the empirically observed dependence on rewarded/unrewarded and common/rare is increased. Meanwhile, the risk-sensitivity axis does not appear correlated with the rare-common stay percentage difference.

While the characteristic pattern of stay percentages can by reproduced by varying parameters *k*^±^ along the uncertainty-sensitivity axis, it can also be reproduced in other ways. Notably, the models used in Daw et al. (2011) show that the characteristic pattern can be reproduced by varying the degree to which their model-based model is used over their model-free model. In addition, a person’s tendency to explore decisions, as reflected in the exploration parameter ∈, could also increase or decrease stay probabilities in our model. In other words, it is difficult to disambiguate a change in how deliberative a person is with their decisions from their ability to learn transitions or their tendency to explore.

### A possible connection to reaction time

Our conceptualization of the Competing-Critics model assumes that the translation of state-action value functions *Q*^+^ and *Q*^–^ into decisions plays out in time, whereby *Q*^+^ and *Q*^–^ determine not only which decisions are made, but also the time until the decision is made, i.e. the reaction time. For example, we hypothesize that *Q*^+^ signals the time at which an action is a viable option to a learner, so that decisions with larger *Q*^+^ are considered earlier. Meanwhile, *Q*^–^ signals the time at which an action is no longer a viable option.

One way to explicitly connect our model to reaction time is to introduce some strictly decreasing function *F*, e.g., *F*(*x*) = exp(-*bx*), that transforms *U_t_*(*a*), which is on the same scale as rewards *R_t_*, to a temporal scale. On trials that the learner behaves greedily (i.e. does not explore), the reaction time could be modeled as min*_a_ F*(*U_t_*(*a*)) with arg min*_a_ F*(*U_t_*(*a*)) determining which action is selected. The probability of selecting a would be left unchanged, since *F* strictly decreasing implies that


\[
\begin{array}{l}
F(\mathop {\max }\limits_a \;{U_t}(a)) = \mathop {\min }\limits_a \;F({U_t}(a))\\
\arg \;\mathop {\max }\limits_a \;{U_t}(a) = \arg \;\mathop {\min }\limits_a \;F({U_t}(a)).
\end{array}
\]

**Figure 8:**
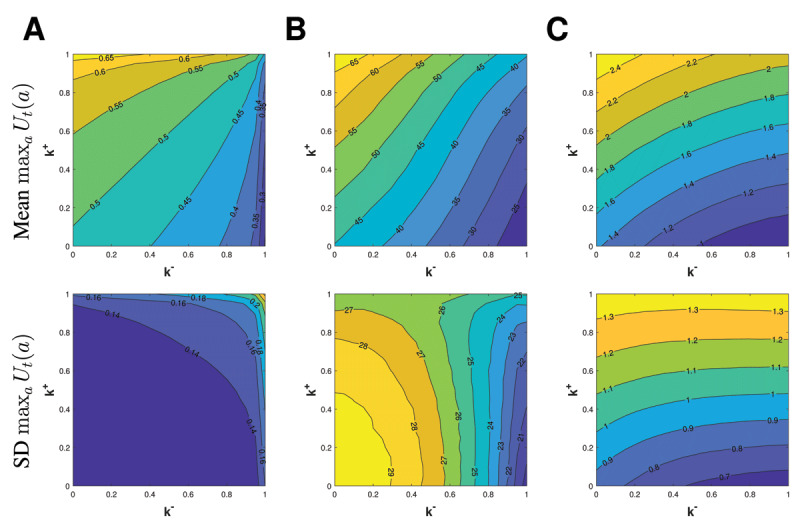
Mean and standard deviation (SD) of max*_a_ U_t_*(*a*) in the **(A)** learning example with *μ* = 0.5, *σ* = 0.2, and skew = 0; **(B)** Iowa Gambling Task; and **(C)** two-stage Markov task. Larger values of max*_a_ U_t_*(*a*) are hypothesized to correspond to faster reaction times.

With the introduction of *F*, there is a one-to-one relationship between reaction time and max*_a_ U_t_*(*a*). Thus, we can learn about reaction times by simulating max*_a_ U_t_*(*a*) for the learning example with *μ* = 0.5, *σ* = 0.2, and skew = 0; the IGT; and the two-stage Markov task (***Figure 8***).

In all three examples, the mean of max*_a_ U_t_*(*a*) varies primarily along the risk-sensitivity axis, with larger values found near (*k*^+^*,k*^–^) = (1,0) and smaller values found near (*k*^+^*,k*^–^) = (0,1). Thus, we would hypothesize that an individual who is risk-seeking would have faster reaction times than an individual that is risk-averse. The standard deviation of max*_a_ U_t_*(*a*), however, does not enjoy a consistent trend. When there is one option available, as in the learning example (***Figure 8A***), the standard deviation of max*_a_ U_t_*(*a*) varies primarily along the uncertainty-sensitivity axis, with larger values found near (*k*^+^*,k*^–^) = (1,1) and smaller values found near (*k*^+^*,k*^–^) = (0,0). This makes sense since the interval (*Q*^+^(*a*),*Q*^–^(*a*)), from which *U_t_*(*a*) is drawn, lengthens when (*k*^+^*,k*^–^) moves towards (1,1). Therefore in this learning example, greater deliberation (i.e. consideration of multiple actions) would not correspond with longer reaction times as one might expect, but rather with greater variability in reaction times. This connection falls apart when there are multiple competing options, with the standard deviation of max*_a_ U_t_*(*a*) varying primarily along the *k*^–^ axis in the IGT and along the *k*^+^ in the two-stage Markov task (***Figure 8B–C***). Thus, we hypothesize that the type of learner who would experience greater variability in reaction times will depend on the task.

Alternatively, our model can be modified to include sequential sampling models, which describe reaction times as first passage times out of some specified region of certain stochastic processes such drift-diffusion models. (Fontanesi, Gluth, Spektor, & Rieskamp, 2019; Kilpatrick, Holmes, Eissa, & Josić, 2019; Lefebvre, Summerfield, & Bogacz, 2020; Veliz-Cuba, Kilpatrick, & Josic, 2016). One possibility is to specify a sequential sampling model for each competing action *a* and select actions according to which corresponds with the fastest first passage times. If one wanted to keep reaction times equal to *F*(*U_t_*(*a*)) and actions selected according to the same probability as our model, then this model would need to be constructed implicitly, so that first hitting times have the same distribution as *F*(*U_t_*(*a*)) with *F* defined above. Otherwise, a preferred sequential sampling model could be specified and state-action values *Q*^±^ used to modulate properties (e.g., drift rate) of this model. This is a common strategy when integrating TD learning with a sequential sampling model.

### Neural encoding of updates

As we mentioned, the rough intuition behind the reinforcement learning update we chose for the state-value functions *Q*^+^ and *Q*^–^ is that they capture the behaviors of risk-seeking and risk-averse learners, respectively. Going even further, we investigate the possibility that dopamine transients encode the update Δ*Q*^+^ associated with the risk-seeking system and serotonin transients encode the negative of the update Δ*Q*^–^ associated with the risk-averse system. In view of this claim, we present one last study, which measured dopamine and serotonin during a decision-making task (Moran et al., 2018).

In this study, participants were asked to make investing decisions on a virtual stock market. In total, participants made 20 investment decisions for 6 markets for a total of 120 decisions. Each participant was allocated $100 at the start of each market and could allocate bets between 0% to 100% in increments of 10%. The participant would gain or lose money depending on their bet. Given a bet *A_t_* on trial *t* and market value *p_t+_*_1_ after betting, percent monetary gain (or loss) on trial *t* was


\[
\left( {\frac{{{p_{t + 1}} - {p_t}}}{{{p_t}}}} \right)\;{A_t}.
\]

To model this experiment, we use the simplifying assumption that bets are low or high: *A_t_ =* {25%, 75%}, and suppose rewards are


\[
{R_t}:\; = \left( {\frac{{{p_{t + 1}} - {p_t}}}{{{p_t}}}} \right)\;\left( {{A_t} - 50} \right).
\]

Actions are centered to 50% to account for the hypothesized role of counterfactual in this experiment (Kishida et al., 2016). Hence, *R_t_* is the percent monetary gain relative to the *counterfactual* gain were a neutral 50% bet made. Following (Moran et al., 2018), trials are split according to a reward prediction error (RPE): the percent monetary gain centered to the mean of its past values and inversely scaled by the standard deviation of its past values.

**Figure 9:**
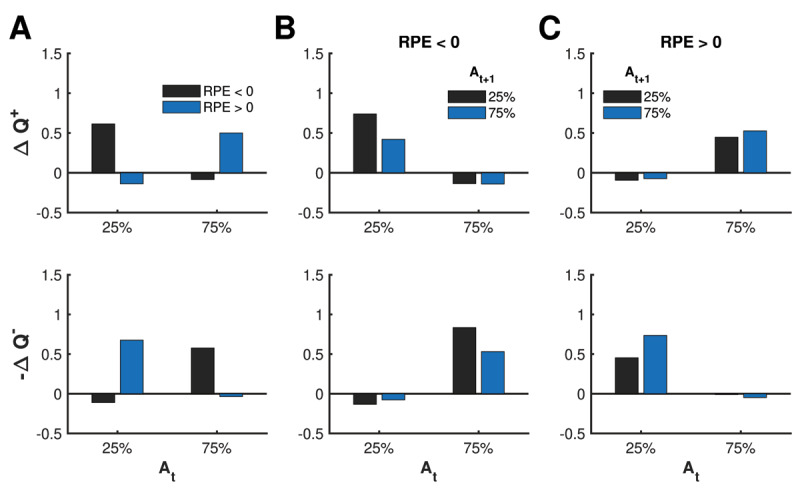
Mean updates as a function of bet levels and reward prediction error (RPE) over 30,000 simulations. **(A)** Mirroring dopamine transients in (Kishida et al., 2016), large mean Δ*Q*^+^ reinforces either a large bet for positive RPE or a small bet when negative RPE. Mirroring serotonin transients in (Moran et al., 2018), large mean *–ΔQ*^–^ reinforces either a large bet for negative RPE or a small bet for positive RPE. **(B–C)** In addition, mean updates can predict the upcoming bet and are asymmetrical, respecting potential asymmetry in the degree to which dopamine and serotonin transients can increase vs. decrease.

Let us consider the scenario where a decision made on trial *t* resulted in a negative RPE, which means the agent has a lower monetary gain relative to expected past gains (***Figure 9A***). Without accounting for counterfactuals, a risk-neutral system would experience a negative update independent of bet level. Risk-seeking update Δ*Q*^+^, however, depends on bet level during a negative RPE: large for a low bet (25%) compared to a high bet (75%). The reverse is true for the negative of the risk-averse update Δ*Q*^–^: it is large for a high bet (75%) compared to a low bet (25%).

These characteristics of Δ*Q*^+^ and -Δ*Q*^–^ during negative RPE mirror, respectively, dopamine and serotonin transients in (Moran et al., 2018). The authors hypothesized that the large dopamine transient for a low bet encourages the *rewarding* decision of betting low, whereas the large serotonin transient for a high bet protects the individual from the *risky* decision of betting high. Betting low is only rewarding when compared to the counterfactual loss of betting a higher amount and losing. This hypothesis is consistent with the role in the Competing-Critics model of a positive Δ*Q*^+^ to encourage a rewarding decision and a negative Δ*Q*^–^ to protect oneself from risky decisions.

When RPE is positive, which is when the agent has a higher monetary gain relative to expected past gains, the direction of the updates flip. The update Δ*Q*^+^ is now large for a high bet (75%) compared to a low bet (25%), and the negative of Δ*Q*^–^ is large for a low bet (25%) compared to a high bet (75%). Again, these characteristics mirror dopamine and serotonin transients in (Kishida et al. 2016; Moran et al., 2018). In this case, it was hypothesized that the relatively large dopamine transient for a high bet encourages the rewarding decision of betting high, whereas the relatively large serotonin transient for a low bet protects the individual from the risky decision of betting low. As before, betting low is only considered risky when compared to the counterfactual loss of what they could have gained if they bet higher.

As an aside, we point out that average updates Δ*Q*^+^ and -Δ*Q*^–^ are generally more positive than they are negative. This asymmetry respects the fact that dopamine and serotonin transients have a biophysical constraint whereby positive transients are easily induced but negative transients are not.

Following (Moran et al., 2018), we consider how updates Δ*Q*^+^ and -Δ*Q*^–^ influence how a person subsequently bets (***Figure 9B–C***). Trials are split further based on the subsequent decision made on the next trial. The negative of update Δ*Q*^–^ is largest when switching from a high to low bet during negative RPE and from a low to high bet during positive RPE. These trends mirror serotonin transients in (Moran et al., 2018), where a relatively large serotonin transient preceded a lowering of a bet when RPE was negative and preceded a raising or holding of a bet when RPE was positive. These findings provided further support that serotonin transients protect an individual from actual and counterfactual losses.

Meanwhile, the update Δ*Q*^+^ is largest when keeping a bet low during negative RPE and when keeping a bet high during positive RPE. Since dopamine transients were not investigated as a function of subsequent bets in (Moran et al., 2018), we have the following hypothesis: a relatively large dopamine transient reinforces a low bet when RPE was negative and reinforces a high bet when RPE was positive.

## Discussion

We presented a computational model of human decision-making called the Competing-Critics model. The model conceptualizes decision-making with two competing critics, an optimist and a pessimist, which are modulated by parameters *k*^+^ and *k*^–^, respectively. We posit that information is integrated from each system over time while decisions compete. The optimist activates decisions (“go”); the pessimist inhibits decisions (“no-go”). We show how our model can illuminate behavior observed in experiments using the Iowa gambling, two-stage Markov, or the stock market tasks.

A key hypothesis of the Competing-Critics model is that the updates in the optimistic and pessimistic learning systems are directly encoded in dopamine and serotonin transients. This finding arose from efforts to reproduce observations during the stock market task in Moran *et al* (Moran et al., 2018) and Kishida *et al* (Kishida et al., 2016). While computational models such as TD learning have provided a useful framework to interpret experiments involving dopamine (Glimcher, 2011), serotonin has been more difficult to pin down (Cools et al., 2011). If serotonin can be understood as updates to a pessimistic learning system, then we would expect serotonin, like dopamine, to influence decision-making in important ways. It would oppose dopamine, protect a person from risky behavior, inhibit certain decisions, and change the value (and timing) of decisions. These functions agree with several leading theories (though not all theories) (Cools et al., 2011; Daw et al., 2002; J. Deakin, 1983; J. W. Deakin & Graeff, 1991; Montague et al., 2016; Moran et al., 2018; Rogers, 2011); yet, the mathematical form we propose for serotonin is new.

We are not the first to try to interpret observations of serotonin and dopamine through the lens of a computational model (Daw et al., 2002; Dayan & Huys, 2008; Montague et al., 2016; Priyadharsini, Ravindran, & Chakravarthy, 2012). Daw *et al*, for instance, describe how prediction error in a TD learning system could be transformed into tonic and phasic parts of dopamine and serotonin signals (Daw et al., 2002). Alternatively, Montague *et al* argue that two prediction errors, derived from reward-predicting and aversive-predicting TD learning systems, could be transformed into serotonin and dopamine transients (Montague et al., 2016). While these models map prediction errors to dopamine and serotonin, the more useful task might be mapping dopamine and serotonin to learning. In other words, trying to understand what certain dopamine and serotonin transients could mean to how a person learns and makes decisions. Our model provides a surprisingly simple answer: dopamine and serotonin transients are exactly the updates to two learning systems.

Critically, these learning systems can capture ranges of decision-making behavior. These learning systems (and hence, dopamine and serotonin) may oppose each other, but they are not perfect antipodes. Hence, the systems are not redundant and obey a principle about efficient coding of information (Montague et al., 2016). For instance, we show that the two learning systems in the Competing-Critics model can implicitly reflect at least two properties of rewards: the mean and standard deviation of rewards. Several other mathematical models of learning and decision-making suggest individuals track the standard deviation of rewards, but do so explicitly (Gershman et al., 2017; Jepma et al., 2020; Li et al., 2011; Redish et al., 2007; Yu & Dayan, 2005).

In addition, the Competing-Critics model reveals how risk-sensitivity and uncertainty-sensitivity represent two orthogonal dimensions of decision-making and how extreme values in either direction could pose unique impairments in decision-making. Sensitivity to risk and uncertainty are well documented in the psychological, economics, and reinforcement learning literature. For instance, risk-seeking (risk-aversion) can be beneficial when large rewards (small losses) are required to escape (avoid) bad scenarios. Platt provides several examples of animals behaving in a risk-sensitive way, e.g., birds switching from risk-aversion to risk-seeking as a function of the temperature (Platt & Huettel, 2008). Miscalibrated risk-sensitivity is thought to cause significant problems for people and underlie a number of psychiatric conditions such as addiction or depression (Korn et al., 2014; Rouhani & Niv, 2019). Mathematically, risk-sensitivity is captured either explicitly through functions that reflect risk-sensitive objectives (Glimcher & Rustichini, 2004; Kahneman & Tversky, 2013) or implicitly through differential weighting of positive and negative prediction errors (Cazé & van der Meer, 2013; Chambon et al., 2020; Hauser et al., 2015; Lefebvre et al., 2017, 2020; Niv et al., 2012; Ross et al., 2018), such as we do here. We recommend the paper by Mihatsch *et al* (Mihatsch & Neuneier, 2002) for a nice theoretical treatment of risk-sensitivity.

Meanwhile, uncertainty-sensitivity represents the degree to which the standard deviation of the reward distribution, and in their knowledge of this distribution, influences their decisions. Like risk-sensitivity, miscalibrated uncertainty-sensitivity is thought to underlie psychiatric conditions such as anxiety (Grupe & Nitschke, 2013; Hirsh, Mar, & Peterson, 2012; Huang, Thompson, & Paulus, 2017; Luhmann, Ishida, & Hajcak, 2011). Huang *et al*, for example, describe this miscalibration in anxiety as a “failure to differentiate signal from noise” leading to a “sub-optimal” decision strategy (Huang et al., 2017). Conceptually, our model provides a different interpretation. Rather than being a failure or sub-optimal behavior, extreme uncertainty-sensitivity embodies a strategy that attempts to satisfy competing objectives, some of which are risk-averse and others which are risk-seeking. In experiments, this conflicted strategy will look similar to an exploration-exploitation trade-off, making it difficult to distinguish between the two.

Interestingly, any attempt to modify solely the optimistic and pessimistic learning system (or dopamine and serotonin transients) will affect both risk sensitivity and uncertainty-sensitivity. The reason is that risk-sensitivity and uncertainty-sensitivity axes are rotated 45 degrees from the axes of the parameters *k*^+^ and *k*^–^ modulating the two learning systems. For instance, increasing *k*^–^ in an attempt to reduce risk-seeking would have the unintended consequence of increasing the sensitivity to uncertainty. Under our interpretation, this would correspond to interventions on serotonin transients to reduce risk-seeking having the potential side-effect of a loss of decisiveness. Similarly, reducing *k*^+^, or intervening on dopamine transients, to reduce risk-seeking would decrease sensitivity to uncertainty. A similar tradeoff occurs when trying to decrease risk-aversion or sensitivity to uncertainty through manipulations of just *k*^+^ or just *k*^–^. Notably, many current pharmacological interventions (e.g., Lithium) act on both dopamine and serotonin neurons.

Another key hypothesis of our model is that values placed on decisions by the two learning system (i.e. *Q*^±^) determine the time to make a decision. Thus, the distribution of reaction time may provide additional data beyond choice selection for which to inform or falsify our model. This connection to reaction time might also help to make sense of the impact of serotonin and dopamine on how quickly decisions are made (e.g., impulsively) (Cools et al., 2011; Niv et al., 2005; Worbe, Savulich, Voon, Fernandez-Egea, & Robbins, 2014). Models for reaction time are often built with stochastic differential equations such as drift-diffusion models to reflect a process of evidence accumulation (c.f., Fontanesi et al. (2019); Kilpatrick et al. (2019); Lefebvre et al. (2020); Veliz-Cuba et al. (2016); for an overview). For example, drift-diffusion models of reaction time can be integrated with a TD learning model by relating drift velocities to different in values between two choices (Pedersen, Frank, & Biele, 2017). Reaction time in our model differs from this approach in that it can arise from any number of possible decisions, as opposed to just two, and is sensitive to risk and uncertainty, rather than a single value, for each decision. This additional flexibility may be useful for explaining experimental observations of reaction time.

There are several limitations of this work to consider. We hope it is clear that the modeling of learning in the updates of *Q*^+^ and *Q*^–^ is largely modular from the modeling that maps these values to actions and reaction times. There are numerous ways that pairs of *Q*^+^ and *Q*^–^ values can be mapped to a choice of actions and a time delay in making that choice. In addition, our model was built upon a Q-learning algorithm, but SARSA-learning may prove to be equally suitable. It should also be clear that our model is over-simplified. One notable absence, for example, is that our model did not track average outcomes or map these outcomes or other parts of our model to tonic dopamine and serotonin, unlike the model of Daw *et al* (Daw et al., 2002). Relatedly, we directly incorporated counterfactuals into our rewards to reproduce findings from the stock market task (Kishida et al., 2016; Moran et al., 2018), but perhaps a separate process, such as tonic serotonin or dopamine, should be included to track counterfactuals. Another limitation of our model is that it relies on only two prediction errors. However, a recent study suggests dopamine is capable of capturing a distribution of prediction errors (Dabney et al., 2020), which has the advantage of being able to learn about the distribution of rewards.

Lastly, one of the key properties of our model, the ordering \[
Q_t^ + > Q_t^ - 
\] assumes that the parameter *α* is the same for *Q*^+^ and *Q*^–^. If parameter *α* were not equal, then the relationship between *Q*^+^ and *Q*^–^ could reverse. The possible effects of *Q*^+^ *< Q*^–^ largely fall outside the specifics of the Competing-Critics model, but it is conceivable such a situation could result in no-go signals arriving before go signals, leading to a decision process unwilling to even consider an option. A situation when no options were even worth consideration may be similar to anhedonia.

In conclusion, this work establishes a new model of human decision-making to help illuminate, clarify, and extend current experiments and theories. Such a model could be utilized to quantify normative and pathological ranges of risk-sensitivity and uncertainty-sensitivity. Overall, this work moves us closer to a precise and mechanistic understanding of how humans make decisions.

## Additional File

The additional file for this article can be found as follows:

10.5334/cpsy.64.s1 Appendix. Supplement for “A competition of critics in human decision-making”.
