# Supplementary material for: A Competition of Critics in Human Decision-Making
Source: Comput Psychiatr. 2021 Aug 12;5(1):81–101. doi: 10.5334/cpsy.64 (PMC11104313; doi:10.5334/cpsy.64)
Supplement: Appendix. — Supplement for “A competition of critics in human decision-making”. [file cpsy-5-1-64-s1.pdf]

Supplement for "A competition of critics in human decision-making"

Enkhzaya Enkhtaivan[1], Joel Nishimura[2], Cheng Ly[3], Amy Cochran[1,4*]

1 Department of Mathematics, University of Wisconsin, Madison, WI, USA

2 School of Mathematical and Natural Sciences, Arizona State University, Glendale, AZ, USA

3 Department of Statistical Sciences and Operations Research, Virginia Commonwealth University, Richmond, VA, USA

4 Department of Population Health Sciences, University of Wisconsin, Madison, WI, USA

* cochran4@wisc.edu

Stochastic Dominance

Let us index $Q^{\pm}$ by time t so that the original update equation will look as follows:

$$Q^{\pm}_{t+1}(S_t, A_t) = Q^{\pm}_t(S_t, A_t) + \alpha\left[(1 \pm k^{\pm})\mathbb{1}_{\delta^{\pm}_t > 0} + (1 \mp k^{\pm})\mathbb{1}_{\delta^{\pm}_t < 0}\right]\delta^{\pm}_t$$

with $Q^{\pm}_0 = 0$ and

$$\delta^{\pm}_t = R_{t+1} + \gamma \max_a Q^{\pm}_t(S_{t+1}, a) - Q^{\pm}_t(S_t, A_t).$$

Proposition 1. *If $k^{\pm} \geq 0$ and $\alpha(1 - k^{\pm}) \leq 1$, then*

$$Q^{+}_t(s, a) \geq Q^{-}_t(s, a)$$

for all pairs $(s, a) \in \mathcal{S} \times \mathcal{A}$ and all non-negative integers t.

Proof. Assume $k^{\pm} \geq 0$ and $\alpha(1 - k^{\pm}) \leq 1$. Our proof is by induction. The base case $t = 0$ is trivial since $Q^{\pm}_0 = 0$. For clarity, let's compute Q_1. In this case, $\delta^{\pm}_1 = R_0$ and so

$$Q^{+}_1(S_0, A_0) = \alpha(1 + k^{+}\text{sign}(R_0))R_0$$

$$Q^{-}_1(S_0, A_0) = \alpha(1 - k^{-}\text{sign}(R_0))R_0.$$

Hence,

$$(Q_1^+ - Q_1^-)(S_0, A_0) = \alpha(k^+ + k^-)|R_0| \geq 0.$$

Now for the inductive hypothesis - assume that $Q_t^+(s,a) \geq Q_t^-(s,a)$ for all pairs $(s,a) \in \mathcal{S} \times \mathcal{A}$ and all non-negative integers up t. One can subtract Q_{t+1}^+ from Q_{t+1}^- to obtain:

$$\begin{aligned}\left(Q^+ - Q^-\right)_{t+1}(S_t, A_t) = &\left(Q^+ - Q^-\right)_t(S_t, A_t) \\ &+ \alpha\left[(1+k^+)\mathbb{1}_{\delta_t^+ > 0} + (1-k^+)\mathbb{1}_{\delta_t^+ < 0}\right]\delta_t^+ \\ &- \alpha\left[(1-k^-)\mathbb{1}_{\delta_t^- > 0} + (1+k^-)\mathbb{1}_{\delta_t^- < 0}\right]\delta_t^-.\end{aligned}$$

Rewrite the above equation in the following form:

$$\begin{aligned}\left(Q^+ - Q^-\right)_{t+1}(S_t, A_t) = &(Q^+ - Q^-)_t(S_t, A_t) \\ &+ \alpha\mathbb{1}_{\{\delta_t^+ > 0 \geq \delta_t^-\}}\left[(1+k^+)\delta_t^+ - (1+k^-)\delta_t^-\right] \\ &+ \alpha\mathbb{1}_{\{\delta_t^+, \delta_t^- > 0\}}\left[(1+k^+)\delta_t^+ - (1-k^-)\delta_t^-\right] \\ &+ \alpha\mathbb{1}_{\{\delta_t^+, \delta_t^- \leq 0\}}\left[(1-k^+)\delta_t^+ - (1+k^-)\delta_t^-\right] \\ &+ \alpha\mathbb{1}_{\{\delta_t^+ \leq 0 < \delta_t^-\}}\left[(1-k^+)\delta_t^+ - (1-k^-)\delta_t^-\right]\end{aligned} \tag{1}$$

So it suffices to check that (1) is non-negative for the four cases for δ_t^-, δ_t^+.

Case 1: $\delta_t^+ > 0 \geq \delta_t^-$. In this case, (1) is trivially non-negative given the non-negativity of $k^\pm$ and the inductive assumption on t.

Case 2: $\delta_t^\pm > 0$. In this case, (1) is a linear function, $L(R_{t+1})$, of R_{t+1}:

$$\begin{aligned}L(R_{t+1}) = &\left(Q^+ - Q^-\right)_t(S_t, A_t) \\ &+ \alpha(k^+ + k^-)R_{t+1} \\ &+ \alpha\gamma\left((1+k^+)\max_a Q_t^+(S_{t+1}, a) - (1-k^-)\max_a Q_t^-(S_{t+1}, a)\right) \\ &- \alpha(1+k^+)Q_t^+(S_t, A_t) + \alpha(1-k^-)Q_t^-(S_t, A_t)\end{aligned}$$

Since L has a non-negative slope $\alpha(k^+ + k^-)$, it stays non-negative if and only if it is non-negative on the

lower bound of its domain. In particular, $\delta_t^{\pm} > 0$ gives lower bounds for R_{t+1}:

$$R_{t+1} > Q^{\pm}(S_t, A_t) - \gamma \max_a Q_t^{\pm}(S_{t+1}, a).$$

If $R_{t+1} = Q^{+}(S_t, A_t) - \gamma \max_a Q_t^{+}(S_{t+1}, a)$, then $\delta_t^{+} = 0$ and

$$\begin{aligned} L(R_{t+1}) &= (Q^{+} - Q^{-})_t(S_t, A_t) - \alpha(1 - k^{-})\delta_t^{-} \\ &= (1 - \alpha(1 - k^{-}))(Q^{+} - Q^{-})_t(S_t, A_t) + \alpha\gamma(1 - k^{-})\Big(\max_a Q_t^{+}(S_{t+1}, a) - \max_a Q_t^{-}(S_{t+1}, a)\Big) \geq 0 \end{aligned}$$

because of the conditions on k^{-} and the inductive hypothesis which is that $Q_t^{+}(s, a) \geq Q_t^{-}(s, a)$ for all pairs. If $R_{t+1} = Q^{-}(S_t, A_t) - \gamma \max_a Q_t^{-}(S_{t+1}, a)$, then $\delta_t^{-} = 0$ and

$$L(R_{t+1}) = (Q^{+} - Q^{-})_t(S_t, A_t) + \alpha(1 + k^{+})\delta_t^{+} \geq 0$$

because of $\delta_t^{+} > 0$ and the inductive hypothesis.

Case 3: $\delta_t^{+}, \delta_t^{-} \leq 0$. Continuing along the similar line of reasoning, one can see that (1) is a linear function, $L(R_{t+1})$, of R_{t+1} with the non-positive slope $-\alpha(k^{+} + k^{-})$. Therefore, it suffices to check that L is non-negative if and only if it is non-negative at its upper bound. Upper bounds for R_{t+1} follow from $\delta_t^{+}, \delta_t^{-} \leq 0$:

$$R_t \leq Q_t^{\pm}(S_t, A_t) - \gamma \max_a Q_t^{\pm}(S_{t+1}, a).$$

If $R_{t+1} = Q_t^{-}(S_t, A_t) - \gamma \max_a Q_t^{-}(S_{t+1}, a)$, then $\delta_t^{-} = 0$ and

$$\begin{aligned} L(R_t) &= (Q^{+} - Q^{-})_t(S_t, A_t) + \alpha(1 - k^{+})\delta_t^{+} \\ &= (1 - \alpha(1 - k^{+}))\left(Q^{+} - Q^{-}\right)_t(S_t, A_t) + \alpha\gamma(1 - k^{+})\Big(\max_a Q_t^{+}(S_{t+1}, a) - \max_a Q_t^{-}(S_{t+1}, a)\Big) \geq 0 \end{aligned}$$

because of conditions on k^{+} and the inductive hypothesis - $Q_t^{+}(s, a) \geq Q_t^{-}(s, a)$ for all pairs.

If $R_{t+1} = Q_t^{+}(S_t, A_t) - \gamma \max_a Q_t^{+}(S_{t+1}, a)$, then $\delta_t^{+} = 0$ and

$$L(R_{t+1}) = (Q^{+} - Q^{-})_t(S_t, A_t) - \alpha(1 + k^{-})\delta_t^{-} \geq 0$$

immediately due to $\delta_t^{-} \leq 0$ and the inductive hypothesis.

Case 4: $\delta_t^+ \le 0 < \delta_t^-$. This is the hardest case. Observe that (1) is again a linear function, $L(R_{t+1})$, of R_{t+1}. Upper and lower bounds on R_{t+1} can be derived from $\delta_t^+ \le 0 < \delta_t^-$, or more explicitly:

$$Q_t^-(S_t, A_t) - \gamma \max_a Q_t^-(S_{t+1}, a) \le R_{t+1} \le Q_t^+(S_t, A_t) - \gamma \max_a Q_t^+(S_{t+1}, a).$$

Therefore, if $L(R_{t+1})$ is non-negative when evaluated at these bounds, it is non-negative on its domain. At the lower bound, one has $\delta_t^- = 0$ and

$$\begin{aligned} L(R_{t+1}) &= (Q^+ - Q^-)_t(S_t, A_t) + \alpha(1-k^+)\delta_t^+ \\ &= (1-\alpha(1-k^+))(Q^+ - Q^-)_t(S_t, A_t) + \alpha\gamma(1-k^+)\Big(\max_a Q_t^+(S_{t+1}, a) - \max_a Q_t^-(S_{t+1}, a)\Big) \ge 0, \end{aligned}$$

which follows from conditions on k^+ and the inductive hypothesis. At the upper bound, one has $\delta_t^+ = 0$ and

$$\begin{aligned} L(R_t) &= (Q^+ - Q^-)(S_t, A_t) - \alpha(1-k^-)\delta_t^- \\ &= (1-\alpha(1-k^-))(Q^+ - Q^-)_t(S_t, A_t) + \alpha\gamma(1-k^-)\Big(\max_a Q_t^+(S_{t+1}, a) - \max_a Q_t^-(S_{t+1}, a)\Big) \ge 0, \end{aligned}$$

which follows from conditions on $k^\pm$ and the inductive hypothesis.

Thus, by induction, we obtain:

$$Q_t^+(s, a) \ge Q_t^-(s, a),$$

for all pairs $(s, a) \in \mathcal{S} \times \mathcal{A}$ and all non-negative integers t. □

We briefly remark about convergence of Q^+ and Q^-. While the authors expect that $Q^\pm$ converge in distribution under the same step-size condition in preceding proposition, a rigorous proof lies outside the scope of this section. Despite the fact that our model is based heavily on the classical reinforcement learning algorithm Q-Learning, the assumption of constant step-size make it difficult to apply the standard techniques of contraction-mapping used in standard literatures. Both in [1] and [2], one can see that it is crucial to have the condition:

1. $\sum_{t=0}^{\infty} \alpha_t = \infty$
2. $\sum_{t=0}^{\infty} \alpha_t^2 < \infty$

On the other hand, Theory of Iterated Random Functions deal with stochastic iterations with constant step-size. However, this usually comes at the expense of much stricter requirements on the rewards such as i.i.d $\{R_t\}$. Classic references on this subject are [3] and [4].

Python Implementation

Here, we elaborate on the implementation of simulations done in the paper. Since we maintain two tables $Q^{\pm}$, two additional risk-parameters $k^{\pm}$, and a more elaborate action-selection method compared to the traditional Q-learning algorithm, we give the skeleton of our python code below:

```
# Defining the different parameters
epsilon = 0.3
S = 30000
T = 100
alpha = 0.5
gamma = 0.0

# In addition to the classical SARSA parameters
# we have k^+, k^-
k_plus = 0.9
k_minus = 0.9

#Initializing the Q^+, Q^- matrices
Q_plus = np.zeros((m,n))
Q_minus = np.zeros((m,n))

# helper function to choose action according to the Balanced-Sarsa
def choose_action(Q_plus, Q_minus, eps, curr_state):
    # CODE HERE
    ...
    return action

# helper function to update the Q-values
def update (curr_action, curr_state, Q, next_action, next_state, r,k,Q_type):
    # CODE HERE
    ...
# the function to encode the piecewise linear update
def piecewise(TD_error, k, Q_type):
    if Q_type == 'POS':
        if TD_error >= 0:
            return (1+k)*TD_error
        else:
            return (1-k)*TD_error
    elif TD_error >= 0:
        return (1-k)*TD_error
    else:
        return (1+k)*TD_error

# function to take a step in the environment
def step(curr_action, curr_state):
```

```python
    # CODE HERE
    ...
    return reward, next_state

#---------Training here---------
for s in range(S)
    # Initialize the initial state and Q-tables here

    curr_action = choose_action(Q_plus, Q_minus, epsilon, curr_state)

    for t in range(T):
        reward, next_state = step(curr_state, curr_action)
        # select the "look-up" action as per SARSA
        next_action = choose_action(Q_plus, Q_minus, eps)

        # update the Q-values
        update(curr_action, curr_state,Q_plus, next_action, next_state, reward , k_plus, "POS")
        update(curr_action, curr_state, Q_minus, next_action, next_state, reward, k_minus, "NEG")

        curr_action = next_action
        curr_state = next_state
```

First of all, the functions **choose action**() , **update**(), and **piecewise**() are identical in all of the tasks, since they are only dependent on the algorithm.

On the other hand, the **step()** function is specific to each task since it needs to capture the transition probabilities and is thus implemented differently for each task. The full implementations can be found on: `https://github.com/eza0107/Opposite-Systems-for-Decision-Making`

Iowa Gambling Task

In the original Iowa Gambling Task, the rewards followed a fixed, discrete distribution and was reset every 10 trials. For instance for Deck A, the first 10 trials included exactly 5 losses each worth \$250 while every trial also gave \$100 and the 5 losses were random within the 10 trial block. Our implementation modified this by choosing a binary reward from $\{\$100, -\$150\}$ with each option being equally likely for Deck A. This simplifies the reward structure a bit while preserving the loss-frequency and the expected gain over trials.

Two-Stage MDP

For this task, complying with the original study in [5], the rewards received at the end of second-stage choice were altered by Gaussian noise with zero mean and 0.025 standard deviation at the end of each second-stage. On the other hand, the probability transitions between first-stage and second-stage were fixed at $\{\text{common, rare}\} = \{p = 0.7, p = 0.3\}$. The code snippet illustrates this below:

```
def reward_prob(T):
    q = np.zeros((2,2,T))
    q[0:2,0:2,0] = np.array([[0.75,0.75],[0.25,0.25]])

    for t in range(1,T):
        q[:,:,t] = q[:,:,t-1] + np.random.normal(0,0.025,[2,2])
        for i in range(2):
            for j in range(2):
                if q[i,j,t]>=1:
                    q[i,j,t] = 1 - (q[i,j,t] - 1)
                elif q[i,j,t]<=0:
                    q[i,j,t] = -q[i,j,t]
    return q
```

Investment Task

Much like the IGT, we have one, stationary state and two actions here. The only point of importance when it comes to implementation is that we considered the counterfactual error and centered rewards, as mentioned previously:

```
def step(curr_action, curr_state, t, market_change):
    reward = (50*curr_action-25)*market_change[t]
    next_state = curr_state
    return reward, next_state
```

Relation to Opposing Actor Learning (OpAL) model

The OpAL model in [6] is similar to our model in that it uses dual competing learning systems and relates these systems to “go” and ”no-go” signals. Despite these similarities, there are many other meaningful differences between the two models, which we expand on below. Let us first introduce the OpAL model. The OpAL model uses a single critic to place a value on each action $V_t(a)$. This value is updated for the selected action A_t according to

$$V_{t+1}(A_t) = V_t(A_t) + \alpha\delta_t,$$

where

$$\delta_t = R_t - V_t(A_t).$$

We point out that these values do not depend on the state S_t to keep with the original presentation of the OpAL model, but could be easily extended to account for state transitions. In the OpAL model, prediction error δ_t influences two populations of neurons, or “actors”, downstream. These populations are denoted by G to represent a go signal and N to represent a no-go signal and updated according to

$$G_{t+1}(A_t) = G_t(A_t) + \alpha_G G_t(A_t)\delta_t,$$
$$N_{t+1}(A_t) = N_t(A_t) - \alpha_N N_t(A_t)\delta_t.$$

for the selected action A_t with $G_{t+1}(a) = G_t(a)$ and $N_{t+1}(a) = N_t(a)$ for actions a not selected and with $G_0 = N_0 = 1$. For simplicity, we will use $\alpha = \alpha_G = \alpha_R = 0.1$ [6].

There are notable differences between the update for G and N and the updates of our model. The first is that the updates of G and N are proportional to G and N. This feature was argued to both better reflect Hebbian learning, keep G and N positive, and introduce greater asymmetry into the relationship between G and A. Mathematically, however, these updates can be unstable in several ways. First, if $\delta_t > 1/\alpha$ such as would happen if there were large rewards, then N would become negative. In addition, G grows exponentially with positive prediction errors δ_t.

Another key difference is that the OpAL model is relatively insensitive to reward uncertainty compared to our Competing-Critics model. Recall, reward uncertainty plays a central role in our model, since it influences an individual’s sensitivity to both risk and uncertainty when making decisions. To demonstrate this

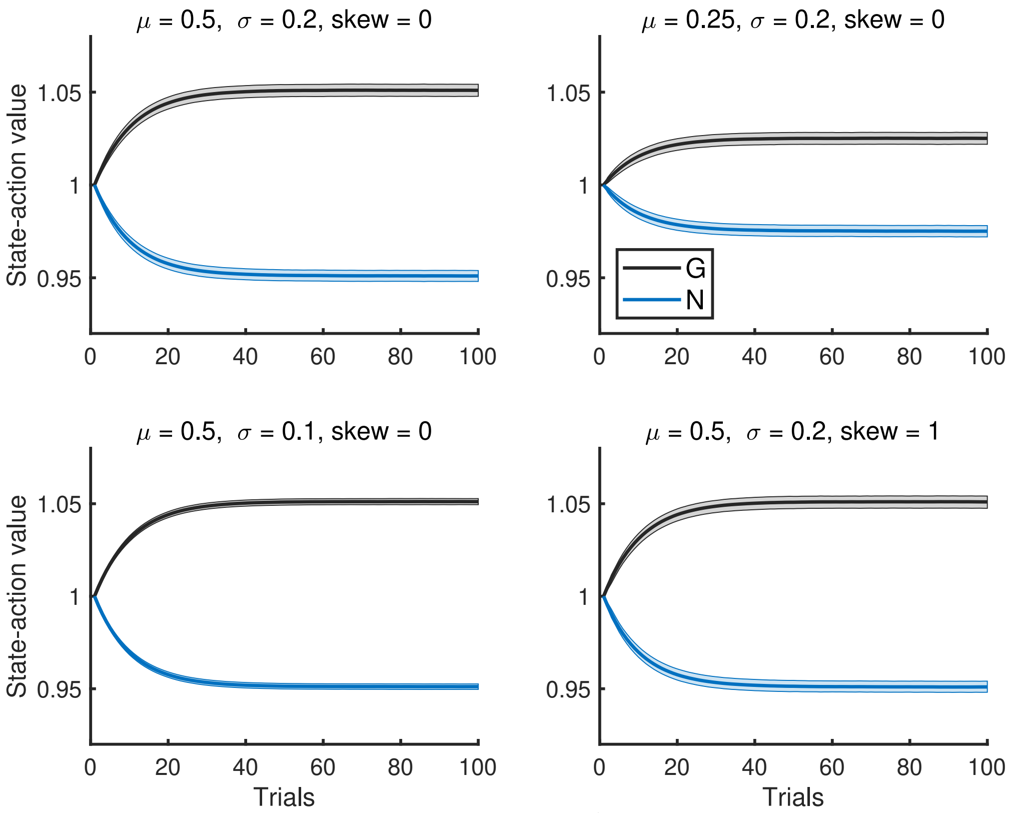

Fig 1. Mean and interquartile range for G and N in the OpAL model.

difference, we simulate the OpAL model on our learning example in the main text (Figure 1). Note that the curves for G and N only change when the mean is changed, in which case the distance between G and N is cut in half. Thus, G and N are insensitive to the standard deviation σ of rewards, whereas $Q^{\pm}$ reflect changes in σ in our model.

When using the OpAL model to make decisions, the authors propose that decision a is selected with probability proportional to

$$e^{\beta_G G(a) - \beta_N N(a)}$$

for constants β_G and β_N. Thus, despite having two values for each decision, only a weighted difference between these values matter when making a decision. This scalarization is also what is related to reaction time in the OpAL model [6]. By contrast, there is no way to transform Q^+ and Q^- into a single value, upon which decisions and reaction times are made in our model.

Further, since G and N are relatively insensitive to reward uncertainty, then decision-making behavior is relatively insensitive to risk. With equal constants $\beta_G = \beta_N = 1$, for example, the OpAL model prefers the

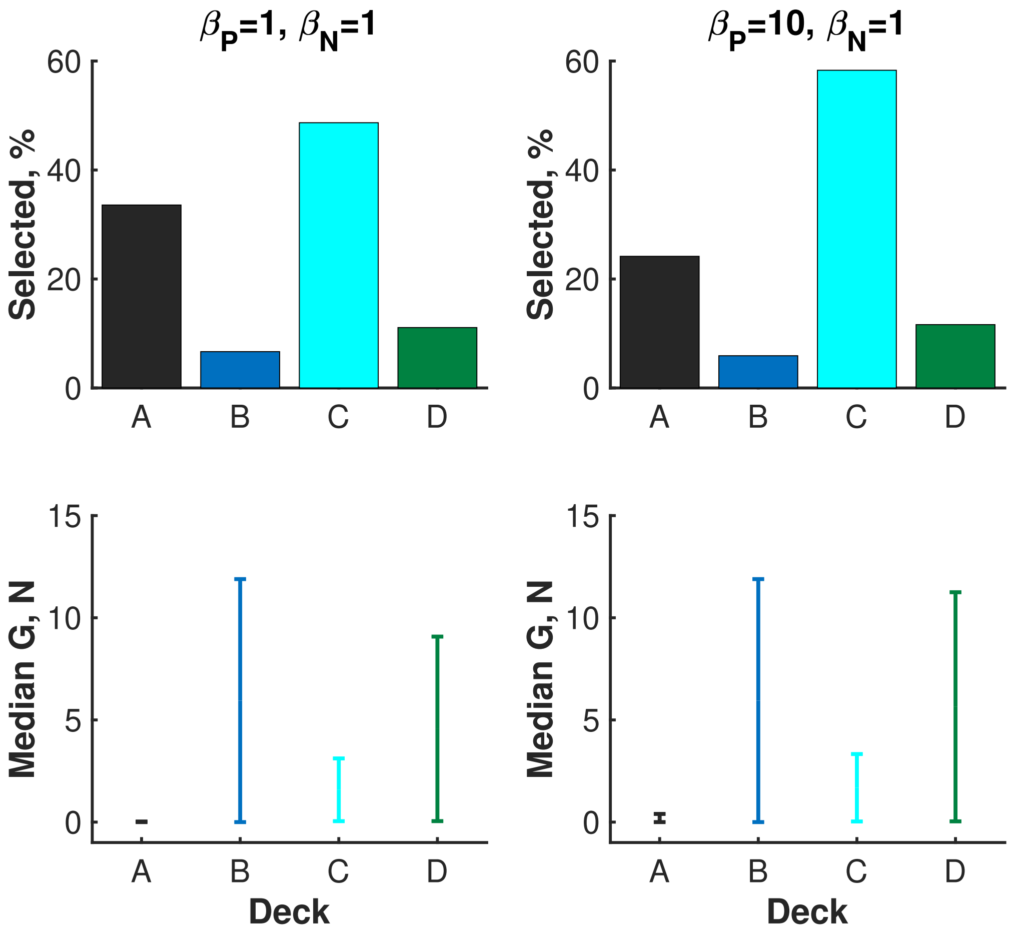

Fig 2. Median values of G and N and probability of desk selection during the Iowa Gambling Task in the OpAL model.

"good" deck C in the Iowa Gambling Task rather than the more risky Decks A and B (Figure 2). Increasing the weight β_G on the "go" population from 1 to 10 only reinforces the choice of Deck C rather than activating more risky decisions.

The authors of the OpAL model posit that prediction error δ_t is still captured by dopamine transients [6], but do not similarly relate serotonin transients to model-derived variable. To investigate whether updates to G or N might reflect serotonin transients (particularly the no-go system N), we simulated the stock market task with the OPaL model (Figure 3). Overall (left panels), the updates G and N mirror the updates $\Delta Q^+, -\Delta Q^-$ in our model and hence, the trends of the dopamine and serotonin transients in the experiment by Moran *et al* [7]. However, when broken down by reward prediction error (RPE), the two models no longer agree. In particular, the update ΔN is not largest when switching from a high to low bet during negative RPE or from a low to high bet during positive RPE. Hence, ΔN does not mirror the trends of serotonin transients in [7], where a relatively large serotonin transient preceded a lowering of a bet when RPE was

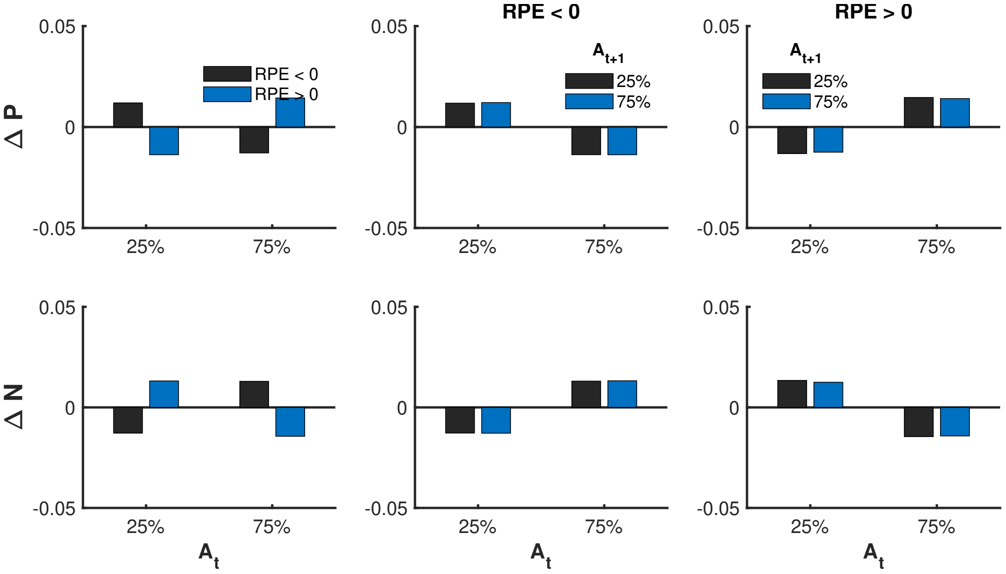

Fig 3. Mean updates of G and N during the stock task in the OpAL model.

negative and preceded a raising or holding of a bet when RPE was positive. Further, the updates are relatively symmetric about zero. In contrast, our model has asymmetric updates, which we hypothesize is easier to encode in a biological system due to physical constraints on firing rates.

In summary, both the OpAL model and our Competing-Critics model use dual learning systems that oppose each other and connect these systems to "go" and "no-go" systems. However, there are a number of notable differences between the models in terms of sensitivity to reward uncertainty and risk, stability, scalarization of dual values to influence decisions and reaction time, and symmetry of updates. For these reasons, we believe the Competing-Critics model contributes a new conceptual framework for human decision-making.

Relation to Risk-Sensitive Model

In constructing the Competing-Critics model, we built off a popular framework known as Risk-Sensitive TD learning. As noted in the main text, this model uses asymmetric updates depending on whether the prediction error is positive or negative:

$$Q(S_t, A_t) \leftarrow Q(S_t, A_t) + \alpha \left[(1+k)\mathbb{1}_{\delta_t>0} + (1-k)\mathbb{1}_{\delta_t<0}\right] \delta_t.$$

where

$$\delta_t = R_{t+1} + \gamma \max_a Q(S_{t+1}, a) - Q(S_t, A_t).$$

We remark that some readers may be more familiar with this exact model but with a different parametrization involving different learning rates: $\alpha^+ := \alpha(1+k)$ and $\alpha^- := \alpha(1-k)$. Decisions are then determined according to the one state-action value model $Q(S_t, a)$.

In terms of learning, the Risk-Sensitive model has only a single value Q to encode information about reward distributions rather than two values Q^+ and Q^- in the Competing-Critics model. More specifically, the value Q is exactly Q^+ when $k = k^+$ for the same sequence of states and actions, and Q^- is left unlearned. Similarly, if $k = -k^-$, then Q coincides with Q^-, and Q^+ is left unlearned. In our learning task illustrated in Figures 3–4 in the main text, the distribution of Q in the Risk-Sensitive model will be exactly the distribution of Q^+ with $k = 0.9$ and the distribution of Q^- with $k = -0.9$. For this task, the Risk-Sensitive model reflects some combination of reward mean and uncertainty, unlike the Competing-Critics model for which the midpoint of $(Q^+ + Q^-)/2$ reflects mean reward and the gap $(Q^+ - Q^-)/2$ reflects reward uncertainty.

In terms of behavior, the Risk-Sensitivity model is designed to modulate risk-sensitivity with the parameter k and does so in a similar way that the parameter gap $(k^+ - k^-)$ does for the Competing-Critics model. This is best illustrated in the Iowa Gambling task (Figure 4). Larger k led risk-seeking behavior with greater preference for Deck B, which can lead to the largest gains but has average net loses. Smaller k led risk-averse behavior with greater preference for Deck C, which can lead to the smallest losses as well as average net gains. These behaviors are reflected in the median Q value over the simulation, with the median Q for each deck decreasing from $k = 0.9$ to $k = -0.9$. At larger k, the median Q reflects the right tails of the reward distribution for the given deck. Hence, Deck B is valued more highly

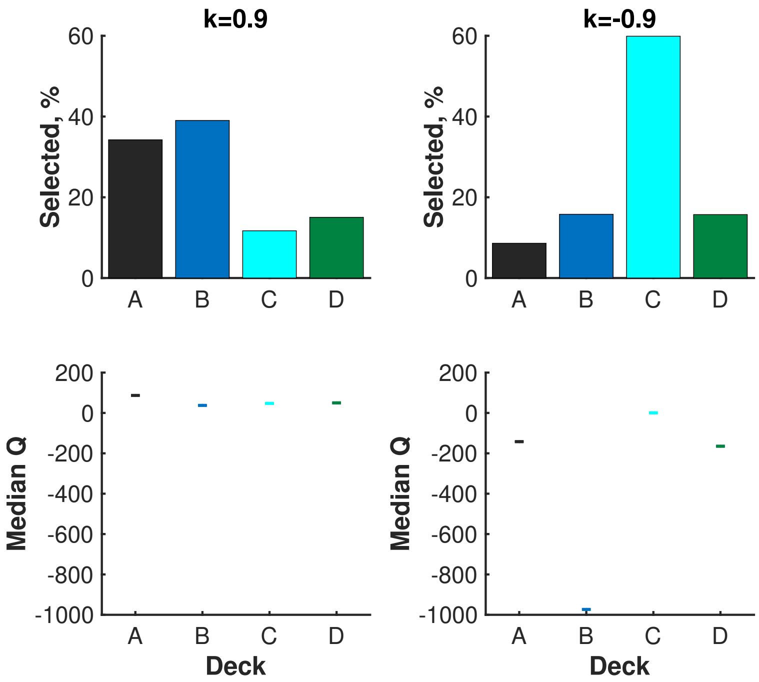

Fig 4. Median values of Q and probability of desk selection during the Iowa Gambling Task in the Risk-Sensitive model.

than the other decks, since it can lead to the largest rewards. At smaller k, the median Q reflects the left tails of the reward distribution for the given deck. Hence, Deck C is valued more highly, since it can lead to the smallest losses.

While the Risk-Sensitive model can modulate risk-sensitive behavior, it cannot also modulate uncertainty-sensitive behavior, since it only has a single value function Q available. With two value functions (i.e. Q^+ and Q^-), the Competing-Critics model modulates uncertainty-sensitive behavior in addition to risk-sensitive behavior with the parameter midpoint $(k^+ + k^-)/2$. This is best illustrated in the two-stage Markov task (Figure 5). Stay probabilities were relatively consistent for different k values in the Risk-Sensitive model. By contrast, stay probabilities (and their gap between common vs. rare transitions) decreased with increasing $(k^+ + k^-)/2$ in the Competing-Critics model, reflecting greater deliberation between choices from a greater emphasis on reward uncertainty.

Finally, we turn to the stock market task (Figure 6). There is only one value Q that is being updated in the Risk-Sensitive model, and so, this update cannot simultaneously reflect both dopamine and serotonin updates. Given that Q can either capture Q^+ or Q^- in the Competing-Critics model depending on the choice of k, then either the update in Q captures the same dopamine trends as the update in Q^+ when k is large, or the negative update in Q captures the same serotonin trends as the negative update in Q^- when k is small. In fact, this observation motivated our construction of the Competing-Critics model out of two different

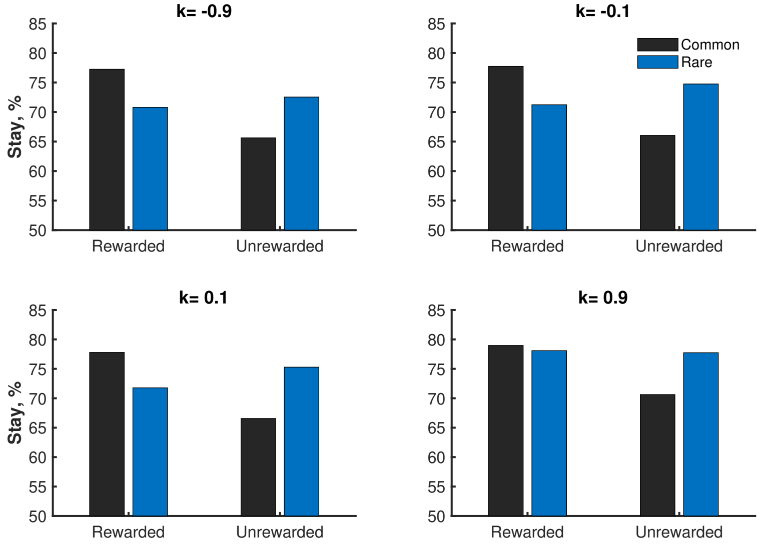

Fig 5. Stay probabilities after a first stage choice over a horizon of 80 decisions (40 first-stage decisions) in the Risk-Sensitive model.

risk-sensitive models. Note also the asymmetry in the updates, which might be more biologically realistic.

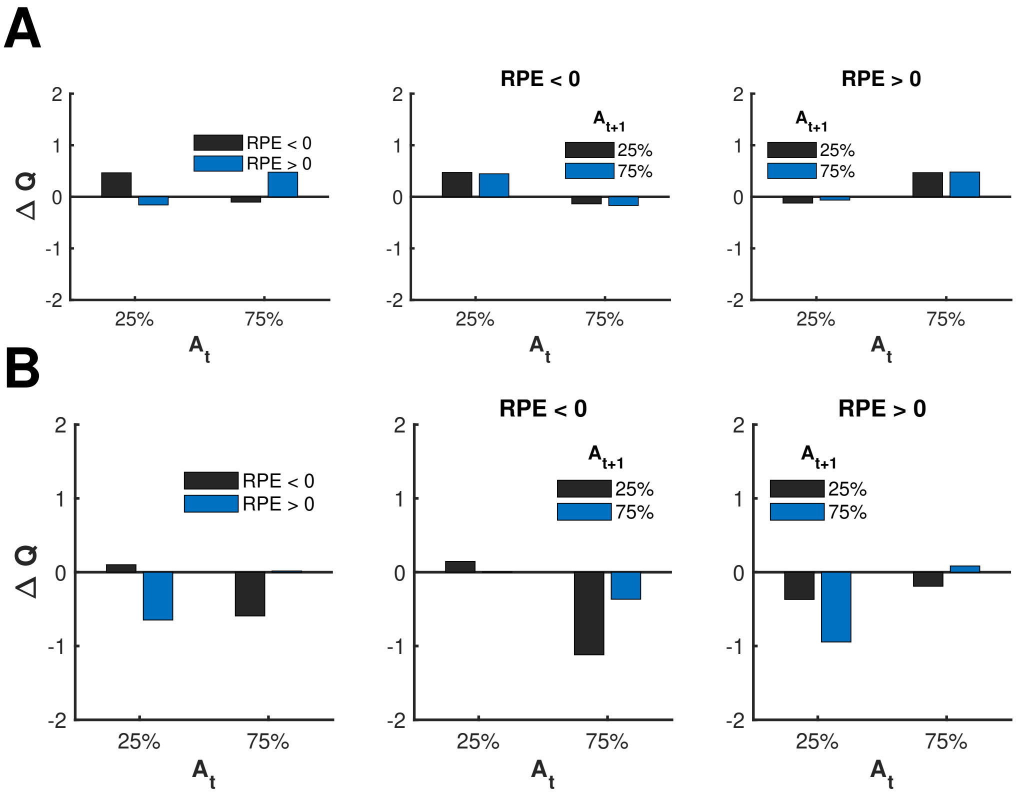

Fig 6. Mean updates as a function of bet levels and reward prediction error (RPE) in the Risk-Sensitive model when **(A)** $k = 0.9$ and **(B)** $k = -0.9$.

Relation to SARSA

We have simulated all the tasks using a SARSA version of our model instead of Q-learning version reported in the main text. As we illustrate below, the SARSA version yields similar behavior to the Q-learning version we included in the main text. So from our simulation experiments, there is little advantage or distinction in preferring Q-Learning to SARSA and vice versa. Consider, for example, the learning example in the main task (Figure 7). The SARSA version of our model leads to similar conclusions: the midpoint and gap of Q^+ and Q^- reflect the mean and standard deviation of the reward distributions.

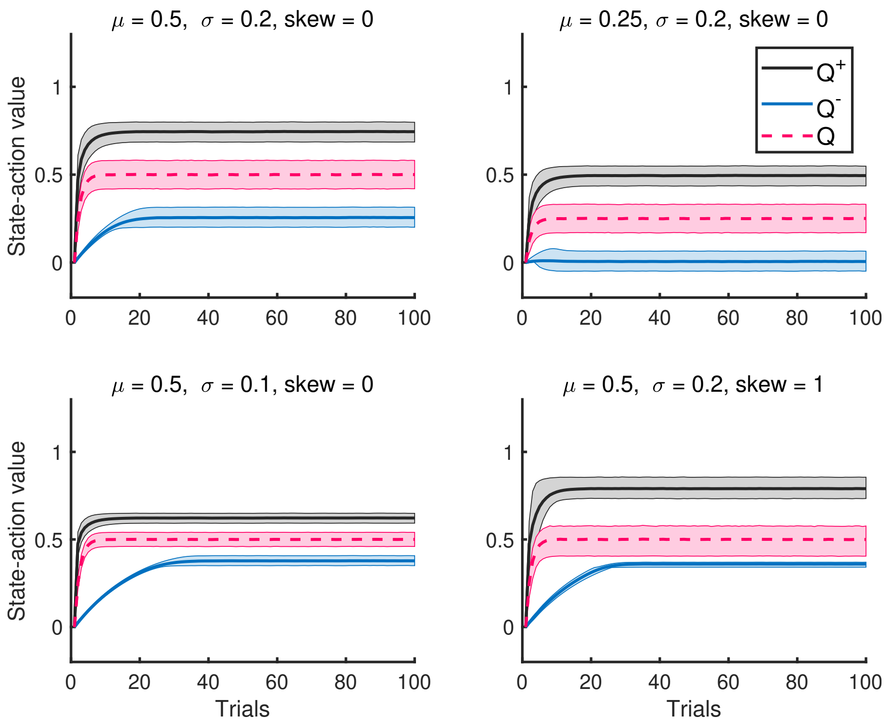

Fig 7. Comparison of mean and interquartile range of state-action value functions over 30,000 simulations in the SARSA version of the Competing-Critics model.

The SARSA version is identical to the model reported in the main text, except that the prediction errors

$$\delta_t^{\pm} = R_{t+1} + \gamma \max_a Q^{\pm}(S_{t+1}, a) - Q^{\pm}(S_t, A_t)$$

are replaced with

$$\delta_t^{\pm} = R_{t+1} + \gamma Q^{\pm}(S_{t+1}, A_{t+1}) - Q^{\pm}(S_t, A_t).$$

For both the Q-Learning and SARSA versions of the model, the TD-error collapses when $\gamma = 0$ to the same update:

$$\delta_t^{\pm} = R_{t+1} - Q^{\pm}(S_t, A_t).$$

Therefore, decision-making would be similar—albeit not identical—in both SARSA and Q-Learning versions for all the tasks we studied in the paper, except the Two-Stage Markov task where we used a non-zero gamma: $\gamma = 0.9$. Decision-making is not identical, because the timing of the update differs, with the SARSA learner updating after action A_{t+1} is selected and the Q-learner updating after transitioning to state S_{t+1} and receiving reward R_{t+1}. In the IGT, for which $\gamma = 0$, the difference in timing yields similar behavior (Figure 8). The preference for good decks versus bad decks varies largely along the risk-sensitive axis, with preference for bad decks when $(k^+, k^-) = (1, 0)$ and preference for good decks when $(k^+, k^-) = (0, 1)$.

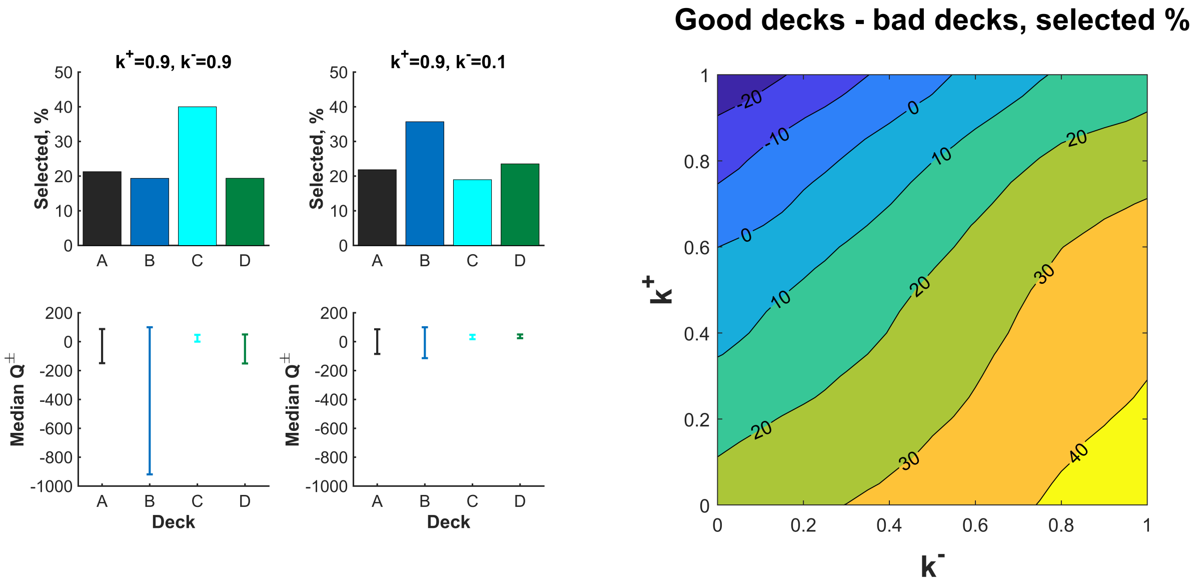

Fig 8. SARSA version of the Figure 6 in the main text for IGT. We see that swapping Q-Learning for SARSA has little effect on the decision-making behavior for the task.

The change in timing of the updates does influence some of the conclusions drawn from the stock market task from [7]. Just like the Q-learning version, the update of ΔQ^+ and $-\Delta Q^-$ in the SARSA version mirror, respectively, dopamine and serotonin transients in [7] during negative and positive RPE (Figure 9A). However, when trials are split further based on the subsequent decision made on the next trial, the negative of update ΔQ^- is largest when keeping a bet high during negative RPE (Figure 9B) and keeping a bet low during positive RPE (Figure 9C). These trends do not mirror serotonin transients in [7], unlike the Q-learning version of the model.

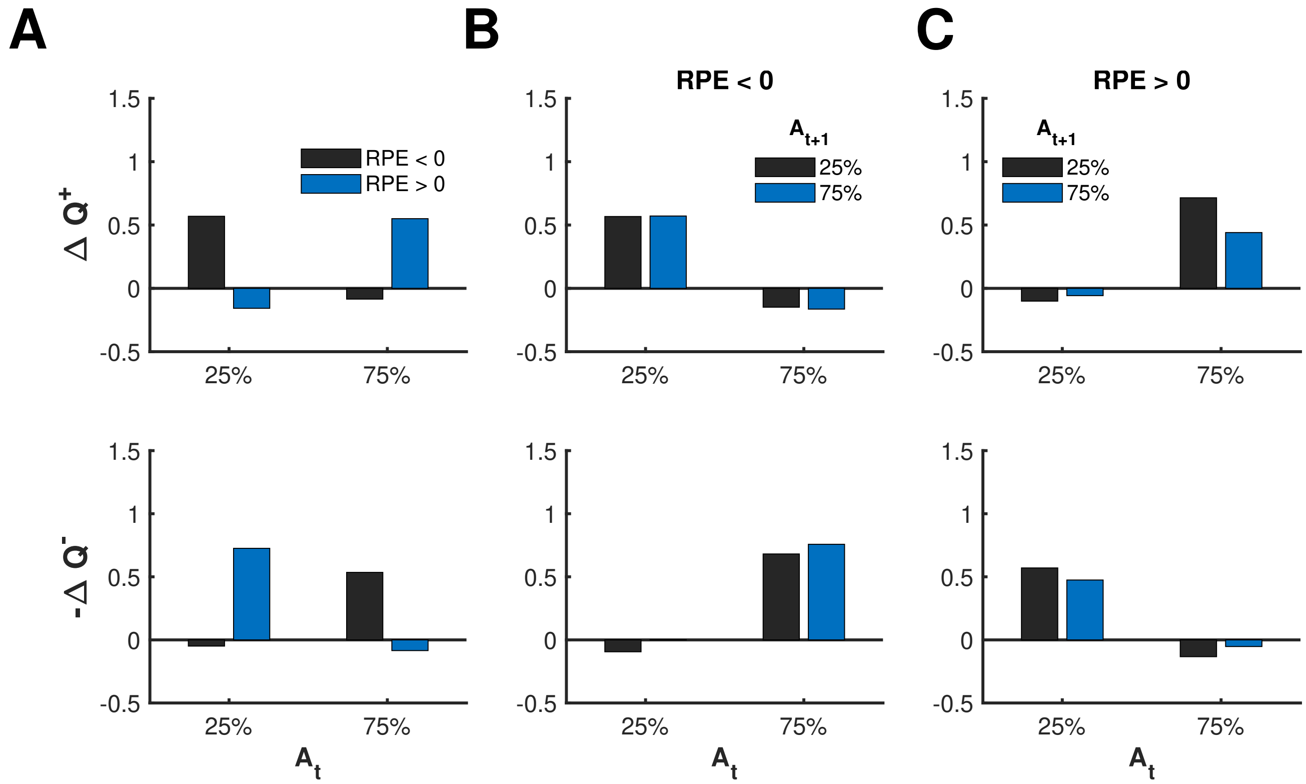

Fig 9. Mean updates as a function of bet levels and reward prediction error (RPE) over 30,000 simulations in the SARSA version of our model.

Even though there are differences in how state-action value functions are updated in the Two-Stage Markov task where we used a non-zero gamma: $\gamma = 0.9$, one can still see from Figure 10 that the behavior under the SARSA version of our model is largely the same as that of the Q-learning version of our model. That is, as $k^{\pm} \to 1$, our learner becomes more deliberative, perhaps as a result of the extreme risk-seeking and risk-averse behaviors balancing each other out, much like the Q version we included in the main draft. Furthermore, when $k^{\pm} \to 0$, the SARSA learner is seen to become rather decisive in choosing their action like the Q-learner.

On the other hand, while the theoretical differences between Q-learning and SARSA may be of interest, our model seemed to benefit little from exploring these distinctions on a deeper level. Perhaps, one argument could be made in favor of SARSA would be that the behavior policy for Q-Learning involves maximizing over several actions $\left(\max_{a \in \mathcal{A}} Q(S_t, a)\right)$ and this may not be ideally suited for studying human decision-making in various settings.

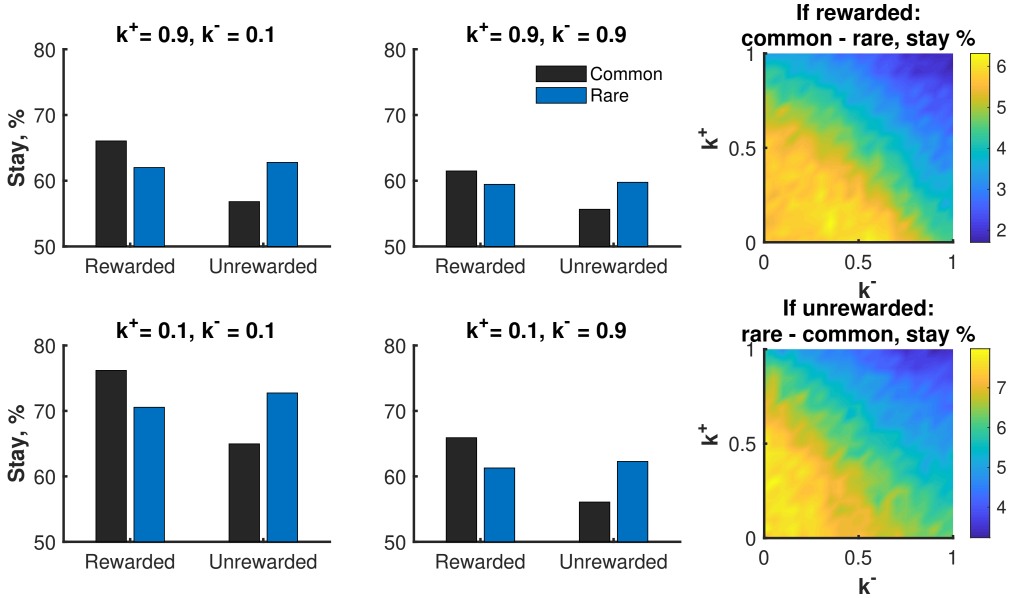

Fig 10. Stay probabilities after a first stage choice over a horizon of 80 decisions (40 first-stage decisions) in the SARSA-learning version of the Competing-Critics model.

Parameters $(\alpha, \varepsilon, \gamma)$ sampled from Beta distribution

Since we fixed a particular set of values of parameters throughout simulations, here we provide additional simulations where we sample them from a Beta distribution with the means preserved:

$$\begin{cases} \alpha \sim \ \text{Beta}(2,2) \\ \varepsilon \sim \ \text{Beta}(0.96, 0.24) \\ \gamma \sim \ \text{Beta}(0.72, 0.08)\,, \ \text{only for Two-Stage task, 0 otherwise.} \end{cases}$$

These choices of Beta parameters ensure:

$$\begin{cases} \mathbb{E}[\alpha] = 0.5, \ \text{Var}[\alpha] = 0.05 \\ \mathbb{E}[\varepsilon] = 0.3, \ \text{Var}[\varepsilon] = 0.05 \\ \mathbb{E}[\gamma] = 0.9, \ \text{Var}[\gamma] = 0.05 \end{cases}$$

Take a look at (Figure 11) below for instance. Just as in the main section (Q-learning) or the preceding section (SARSA-learning), we see that the midpoint and gap of Q^+ and Q^- reflect the mean and standard

deviation of the reward distribution.

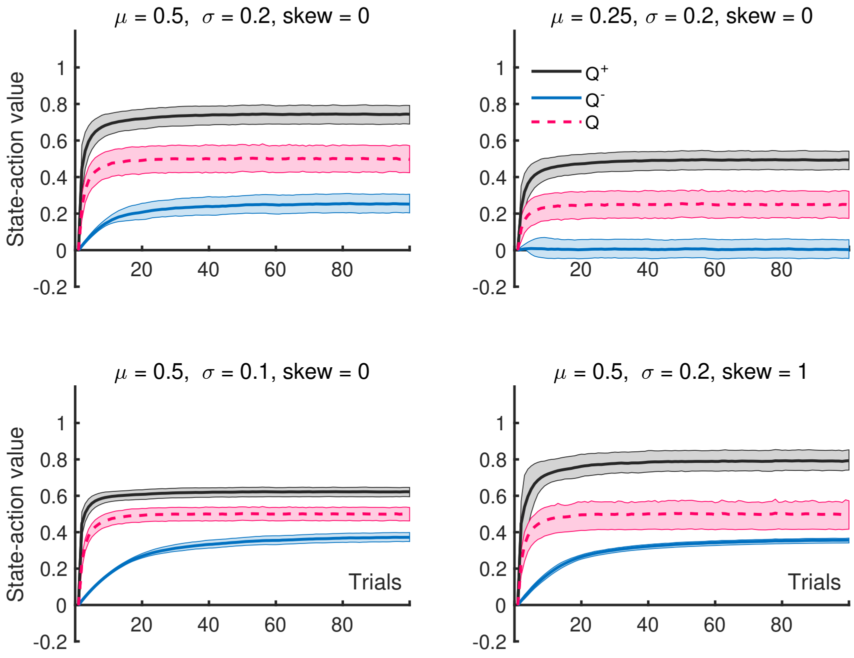

Fig 11. Comparison of mean and interquartile range of state-action value functions over 3000 simulations when α and ε were drawn from the specified Beta distribution.

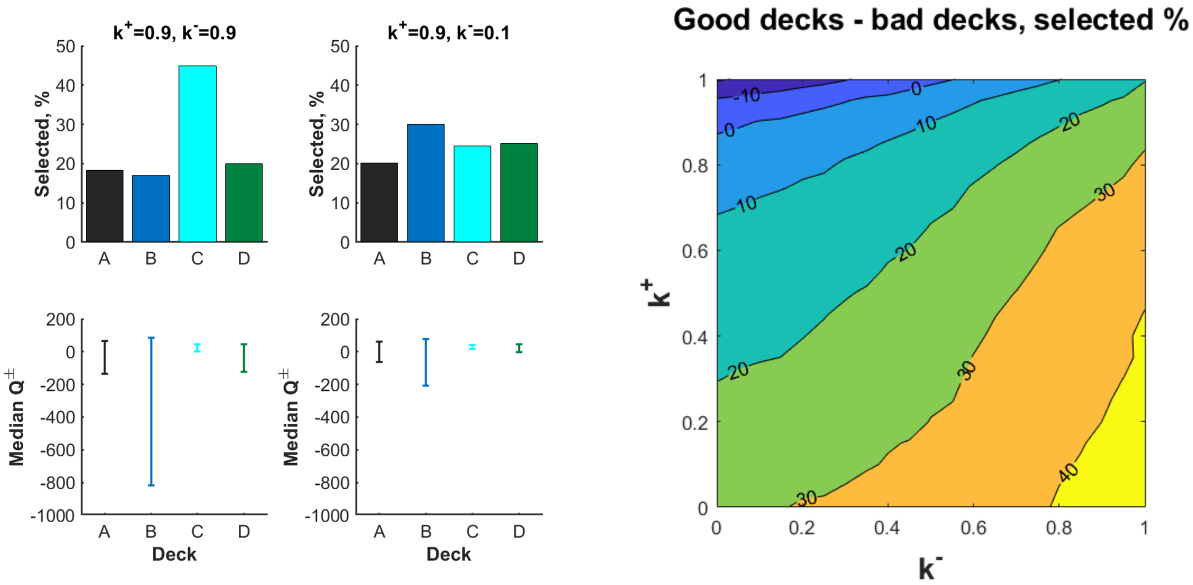

Fig 12. Figure 6 in the main text for IGT, when we sample the parameters α and ε.

As with the fixed parameter version of Competing-Critics model, parameter-sampled version yields the

same behavior for Iowa Gambling Task. Same thing with the Stock Market Task - (Figure 13) shows that the key findings from Figure 9 in the main text still holds. That is, (A) Mirroring dopamine transients in [8], large mean ΔQ^+ reinforces either a large bet for positive RPE or a small bet when negative RPE. Mirroring serotonin transients in [7], large mean $-\Delta Q^-$ reinforces either a large bet for negative RPE or a small bet for positive RPE. (B–C) In addition, mean updates can predict the upcoming bet and are asymmetrical, respecting potential asymmetry in the degree to which dopamine and serotonin transients can increase vs. decrease.

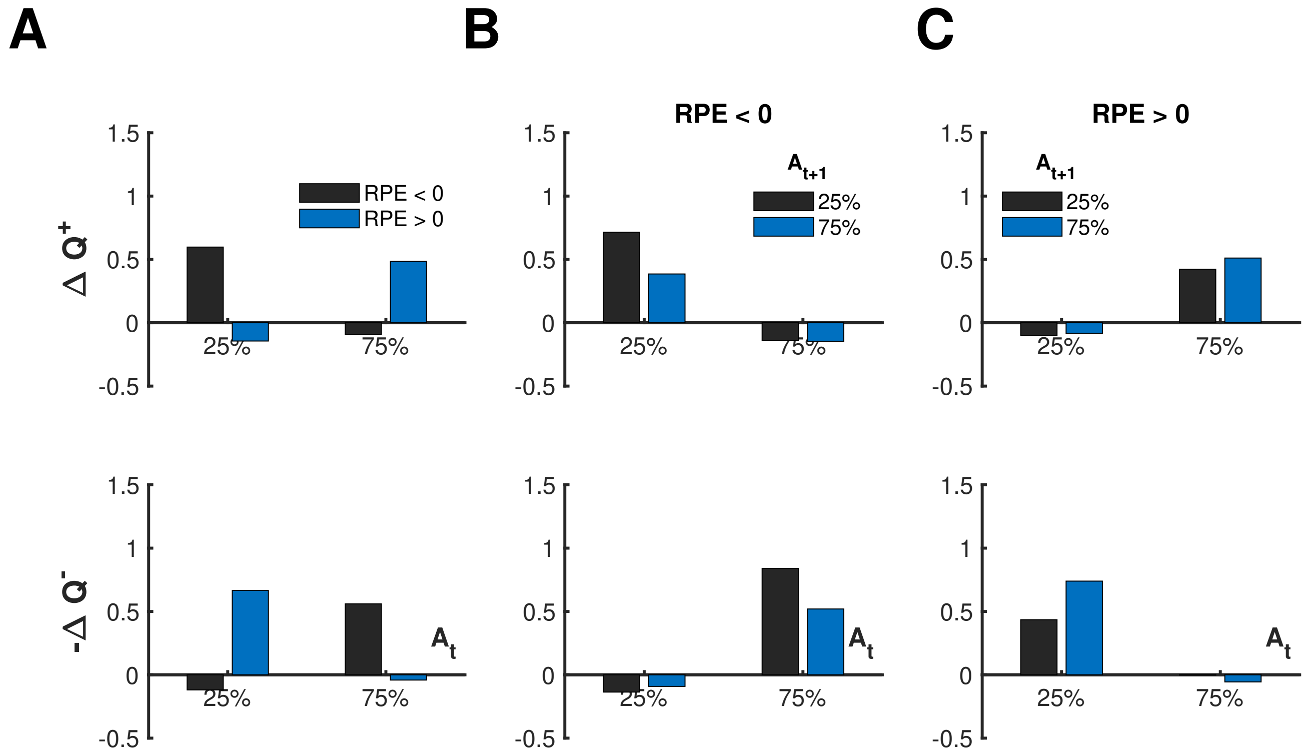

Fig 13. Mean updates as a function of bet levels and reward prediction error (RPE) over 1000 simulations when α and γ are sampled.

Lastly, one can see from (Figure 14) the Two-Stage Markov Task produces the same result when we sample $(\alpha, \varepsilon, \gamma)$ in the Competing-Critics model. The learner becomes more deliberate as $k^{\pm} \to 1$ and more indecisive $k^{\pm} \to 0$, as discussed in the main text under Figure 7.

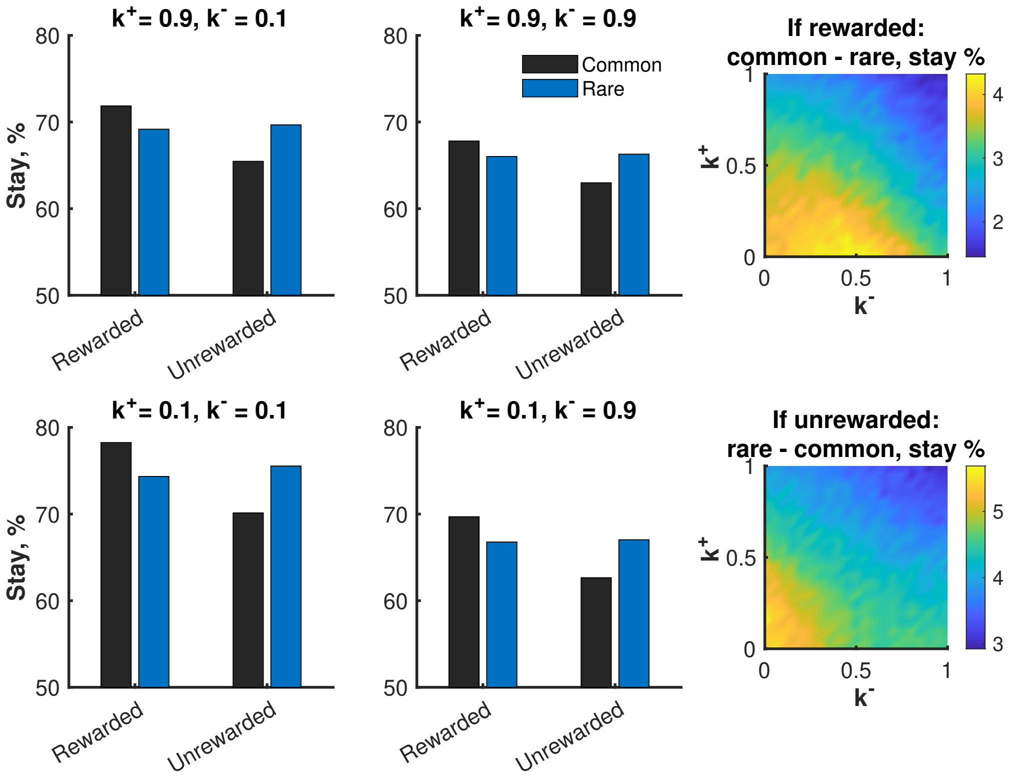

Fig 14. Stay probabilities after a first stage choice over a horizon of 80 decisions (40 first-stage decisions) in the Competing-Critics model when we sample α, γ and ε.

References

1. Watkins CJCH, Dayan P. Q-learning. In: Machine Learning; 1992. p. 279–292.
2. Mihatsch O, Neuneier R. Risk-sensitive reinforcement learning. Machine learning. 2002;49(2-3):267–290.
3. Diaconis P, Freedman D. Iterated random functions. SIAM Review. 1999;41:45–76.
4. Duflo M. Random Iterative Models. Springer Berlin Heidelberg; 1997. Available from: https://doi.org/10.1007%2F978-3-662-12880-0.
5. Daw ND, Gershman SJ, Seymour B, Dayan P, Dolan RJ. Model-based influences on humans' choices and striatal prediction errors. Neuron. 2011;69(6):1204–1215.
6. Collins AG, Frank MJ. Opponent actor learning (OpAL): Modeling interactive effects of striatal dopamine on reinforcement learning and choice incentive. Psychological review. 2014;121(3):337.
7. Moran RJ, Kishida KT, Lohrenz T, Saez I, Laxton AW, Witcher MR, et al. The protective action encoding of serotonin transients in the human brain. Neuropsychopharmacology. 2018;43(6):1425.
8. Kishida KT, Saez I, Lohrenz T, Witcher MR, Laxton AW, Tatter SB, et al. Subsecond dopamine fluctuations in human striatum encode superposed error signals about actual and counterfactual reward. Proceedings of the National Academy of Sciences. 2016;113(1):200–205.
